# Fungal-like particles and macrophage-conditioned medium are inflammatory elicitors for 3T3-L1 adipocytes

**DOI:** 10.1038/s41598-020-66283-4

**Published:** 2020-06-10

**Authors:** Chanawee Jakkawanpitak, Nongporn Hutadilok-Towatana, Decha Sermwittayawong

**Affiliations:** Department of Biochemistry, Faculty of Science, Prince of Songkla University, Hat Yai, 90110 Thailand

**Keywords:** Polysaccharides, Acute inflammation, Stress signalling

## Abstract

Adipocytes from white-adipose tissue are known to produce inflammatory cytokines, which play a major role in energy balance and metabolism. While they can respond to pathogen-associated molecular pattern (PAMPs) such as lipopolysaccharide (LPS) from bacteria, it is not known whether adipocytes can be stimulated by fungal cells. Previously, adipocytes were shown to produce toll-like receptor 2 (TLR2), a β-glucan receptor, suggesting that they could respond to β-glucan on the fungal cell wall. In this study, we show that heat-killed yeast induce an inflammatory response in adipocytes. Using fungal-like particles, namely laminarin-coated beads (LCB), we find that these particles trigger the expression of many key inflammatory genes in dose- and time-dependent fashions in adipocytes. These results suggest that β-glucan on the fungal cell wall is sufficient to elicit an inflammatory response in adipocytes. In addition, we show that both LCB and LCB-treated conditioned medium from RAW 264.7 murine macrophages (LCB-RM) induce the expression of those inflammatory genes through IKKβ-IκBα proteins. Together, we conclude that the fungal-like particles and the conditioned medium elicit an inflammatory response in adipocytes through the canonical or classical NF-κB pathway.

## Introduction

Improper balance between excessive food intake and adipose tissue regulation results in obesity, which is a major epidemic problem worldwide. Obesity is considered an inflammatory condition accompanied by an increase in the levels of circulating proinflammatory cytokines. Obesity is an independent risk factor for several diseases such as insulin resistance, type-2 diabetes, cardiovascular disease, hypertension and stroke, arthritis, and certain forms of cancer^1^. The increase in circulating inflammatory cytokines without clinical signs of inflammation is referred to as *low-grade inflammation*^2^.

Adipose tissue, a complex organ that consists of many cell types such as adipocytes and immune cells, is a major contributor to many adipokines, inflammatory cytokines and chemokines. The increase in circulating inflammatory cytokines during low-grade inflammation can be caused by several mechanisms, one of which involves the collaboration between adipocytes and immune cells in adipose tissue. A previous study showed that monocyte chemoattractant protein-1 (MCP-1/CCL2) is highly produced in the adipose tissue from genetically obese diabetic (leptin receptor-deficient or *db*/*db*) and wild-type mice with obesity induced by a high-fat diet^3^. MCP-1/CCL2 is recognized by the C-C chemokine type 2 (CCR2) receptor, which is expressed on the surface of monocytes^4^. The presence of MCP-1/CCL2 attracts macrophages to infiltrate the adipose tissue^3^. These are the M1-polarized macrophages^5^, which produce and release inflammatory mediators such as tumornecrosis factor alpha (TNF-α), interleukin-1β (IL-1β), and IL-6. The chronic increase in these inflammatory cytokines causes the impairment of insulin signaling in the insulin-sensitive organs such as adipose tissue and skeletal muscles. For example, it was shown that IL-6 reduces the expression of glucose transporter 4 (GLUT-4) and insulin receptor substrate 1 (IRS-1) genes in 3T3-L1 adipocytes^6^. The defect in the insulin signaling pathway in adipose tissue and skeletal muscles reduces their sensitivity to insulin, leading to the development of insulin resistance. Consistent with this notion, mice with obesity induced by a high-fat diet develop insulin resistance^3^.

In addition to the chronic inflammation caused by macrophage infiltration, lipopolysaccharide (LPS) plays an important role in the inflammation of adipose tissue. LPS, which is located in the outer cell membrane of Gram-negative bacteria, is released when bacteria die and is prevented from translocation into the bloodstream by the intestinal epithelium^7^. However, this protective function of the intestinal epithelium is impaired in obese animals fed with a high-fat diet. It was shown that fatty acids in the high-fat diet increased the number of Gram-negative bacteria and permeability of the intestinal epithelium, resulting in an increasing concentration of LPS in the circulation^8^. Thus, the high-fat diet from food caused the rise in circulating LPS. A state of chronic low-grade inflammation induced by systemic exposure to bacterial LPS is referred to as *diet-induced metabolic endotoxemia*^9^. Consistently, mice fed with a high-fat diet for 4 weeks showed an increase in plasma LPS by 2-or 3-fold, body fat, and expression of inflammatory cytokines and developed insulin resistance^10^.

The mechanism of how LPS induces an inflammatory response in macrophages and adipocytes is well characterized. Both adipocytes and macrophages are capable of recognizing LPS through the Toll-like receptor 4 (TLR4)^11^. LPS stimulated macrophages to produce a number of inflammatory cytokines such as TNF-α, interleukin 1 beta (IL-1β) and inducible nitric oxide synthase (iNOS)^12^. These inflammatory cytokines are sensed by other cells, including adipocytes, which subsequently respond by producing other inflammatory cytokines such as IL-6, MCP-1/CCL2, and cyclooxygenase 2 (COX-2)^13^. In addition, LPS was shown to stimulate an inflammatory response in adipocytes both *in vivo* and *in vitro* ^14,15^. It was shown that LPS induces an inflammatory response in 3T3-L1 adipocytes through inhibitor of IκB kinase β (IKKβ) protein which promotes proteasomal degradation of inhibitor of IκB alpha (IκBα) protein^14^, suggesting the involvement of the canonical/classical NF-κB pathway^16^.

Although the mechanistic effect of LPS from Gram-negative bacteria on stimulating an inflammatory response in adipocytes is well understood, it is not known whether β-glucan from pathogenic fungi could induce such a response in fat cells. β-glucan can be recognized by several receptors on the cell surface, including the Toll-like receptor 2 (TLR2)^17^. Interestingly, previous research showed that treatment of 3T3-L1 adipocytes with either LPS, zymosan or TNF-α stimulated a production of TLR2 receptor, which does not normally express in the untreated cells^18^. Additionally, the analysis of gene expression profile in adipose tissue isolated from mice with acute systemic inflammation induced by injection with LPS showed changes in the expression of over 6,000 genes, including the upregulation of TLR2^15^. These studies suggest that adipocytes have the ability to recognize and respond to β-glucan on the fungal cell wall. However, no study so far has tested whether adipocytes produce an inflammatory response to β-glucan or fungal particles. In order for β-glucan to stimulate an inflammatory response, at least in the context of immune cells, it must be in a particulate form or immobilized on beads or a plastic surface^19–22^. Thus, it is possible that adipocytes may respond to β-glucan just like immune cells.

Those studies led us to hypothesize that the adipocytes produce an inflammatory response to fungal particles and that the immobilized β-glucan is sufficient to induce such a response. Therefore, we tested the ability of the heat-killed fungal cells to stimulate an inflammatory response to 3T3-L1 adipocytes, which is used as a model for adipocytes. In addition, the mechanism of 3T3-L1 adipocytes to produce an inflammatory response against polystyrene beads coated with laminarin, namely laminarin-coated beads (LCB) and the conditioned medium from RAW 264.7 murine macrophages treated with LCB is analyzed and discussed in this study.

## Results

### Heat-killed fungal particles induce an inflammatory response in 3T3-L1 adipocytes

**Figure 1:**
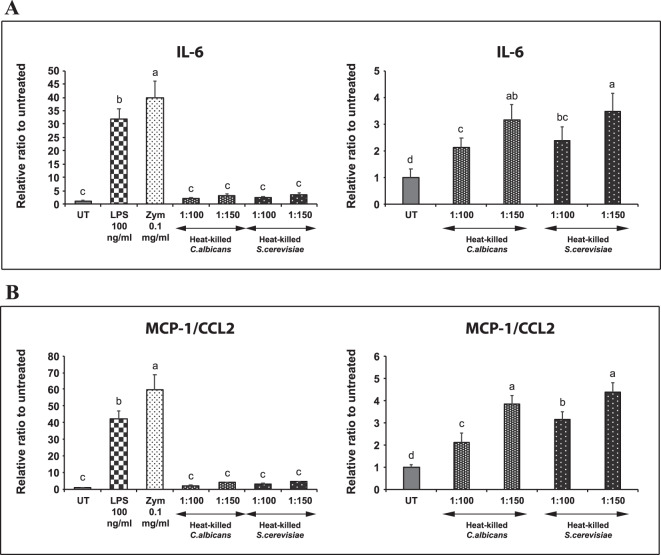
Heat-killed yeast stimulates the expression of inflammatory genes in differentiated adipocytes. (**A**) shows IL-6 gene expression in differentiated adipocytes treated for 3 hours with 100 ng/mL lipopolysaccharide (LPS), 0.1 mg/mL zymosan (Zym), or the increasing amount of heat-killed *C. albicans* or *S. cerevisiae*. LPS is a positive control. UT is abbreviated for untreated. The right panel is the same dataset as in the left panel, except that the results from LPS and zymosan are omitted. 1:100 and 1:150 are the ratios of adipocytes to yeast particles. (**B)** is similar to (**A**), except that the expression of MCP-1/CCL2 was analyzed.

The inducible TLR2, a β-glucan receptor in 3T3-L1 adipocytes led us to investigate the ability of the cells to produce an inflammatory response against the heat-killed fungal particles. The expression levels of IL-6 and MCP-1/CCL2 in differentiated 3T3-L1 adipocytes stimulated with LPS, zymosan, or an increasing amount of heat-killed *Saccharomyces cerevisiae* or *Candida albicans* were analyzed with quantitative PCR (qPCR). LPS was included as a positive control because it was shown to stimulate an inflammatory response in 3T3-L1 adipocytes^14^. The results show that the IL-6 expression in the heat-killed-stimulated adipocytes is relatively low compared with the LPS- or zymosan-stimulated cells (Fig. 1A, left panel). However, when the data set from LPS and zymosan is omitted, both heat-killed *S. cerevisiae* and *C. albicans* significantly induce the IL-6 gene expression in a dose-dependent manner (Fig. 1A, right panel). Likewise, the heat-killed yeasts also dose-dependently stimulate MCP-1/CCL2 gene expression (Fig. 1B).

The fungal cell wall contains β-glucans and other components such as proteins, lipids, mannoprotein, and chitin^23,24^. The presence of other cell wall components probably limits the accessibility of adipocytes to β-glucan. In contrast, zymosan, which is made from *S. cerevisiae*, has a low protein content and a high ratio between β-glucan and mannan^25^. This likely explains why the levels of gene expression in the zymosan-treated cells are much higher than those in the heat-killed yeasts-treated adipocytes. Another possibility is that a component in the cell wall of yeast may inhibit the recognition or responses by the receptor on adipocytes. A previous study demonstrated that chitin in the inner cell wall of fungal pathogens indirectly blocked the recognition of β-glucan by the Dectin-1 receptor on human peripheral blood mononuclear cells (PBMCs) and murine macrophages^26^. However, it is not known whether Dectin-1 is the β-glucan receptor on the surface of adipocytes. Despite a low level of gene expression, this experiment illustrates the ability of differentiated adipocytes to produce an inflammatory response to the actual fungal particles.

### LCB efficiently induces an inflammatory response in RAW 264.7 murine macrophages

The results from Fig. 1 led us to further hypothesize that β-glucan, which is present in the cell wall of fungal cells, triggers an inflammatory response in 3T3-L1 adipocytes. To test this hypothesis, we created β-glucan-coated beads as fungal-like particles, which were synthesized using the same protocol previously described by Tam and colleagues^20^. Because it was previously shown that the conjugated beads triggered an inflammatory response in macrophages just like the fungal cells^20^, β-glucan-coated polystyrene beads can be called “fungal-like particles”. Furthermore, these β-glucan-coated polystyrene beads do not contain proteins or other polysaccharides from the fungal cells, excluding the possibility that the inflammatory effect is caused by factors other than the β-glucan.

We chose laminarin, a small soluble β-glucan from *Laminaria digitata*, as a source of β-glucan to produce laminarin-coated beads (LCB). Quantification of the amount of the polysaccharides on the beads revealed that the average concentration of laminarin on the beads was 1.1 × 10^–7^ μg/beads. To examine whether our newly created LCB was functional, we first tested its toxicity effect and the ability to induce an inflammatory response in RAW 264.7 murine macrophages. The MTT assay reveals that the percent viability of the RAW 264.7 cells decreases as the cells:particles ratio increases, and the maximum ratio of 1:30 cells:particles allows 80% viability (Fig. S1). We predict that the viability of the cells would decrease further if the ratio were to be increased beyond 1:30. Thus, this ratio of 1:30 cells:LCB was used as the maximum concentration with the RAW 264.7 cells.

**Figure 2:**
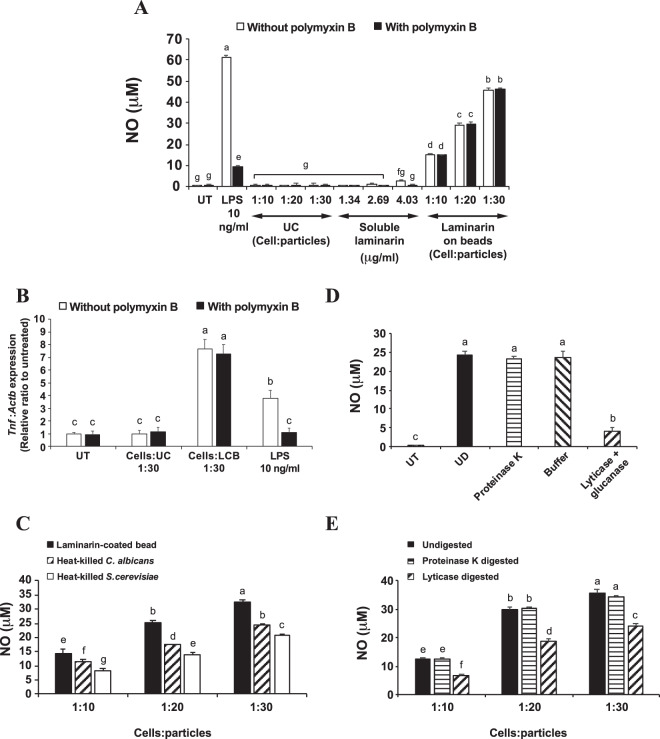
LCB induces an inflammatory response in RAW 264.7 murine macrophages. (**A**) shows the level of nitric oxide (NO) produced by RAW 264.7 murine macrophages after 24-hour post-stimulation with various concentrations of laminarin (soluble vs conjugated beads) and with or without 10 μg/mL polymyxin B added. Lipopolysaccharides (LPS) were included as a positive control for NO production and polymyxin B test. UT and UC are abbreviated for untreated and unconjugated beads, respectively. (**B)** shows the expression of TNF-α gene in RAW 264.7 murine macrophages treated for 6 hours with the uncoated beads (UC), LCB or LPS at 10 ng/mL, in the presence or the absence of 10 μg/mL polymyxin B. The expression was measured using quantitative PCR (qPCR). (**C)** shows the amount of NO released from RAW 264.7 murine macrophages treated with LCB and heat-killed *C. albicans* or *S. cerevisiae* at 3 different ratios (cells:particles) for 24 hours. (**D)** shows the amount of NO released from RAW 264.7 murine macrophages treated for 24 hours with either LCB or polystyrene beads conjugated with proteinase K-digested, sodium acetate buffer pH 5.0-treated or lyticase + glucanase-digested laminarin (pre-digested laminarin before conjugation). The ratio between cells and LCB was at 1:30. (**E**) is like (**D**), except that the digestion was performed with LCB (digestion after conjugation).

The ability of LCB to induce an inflammatory response in the RAW 264.7 cells was accessed by detecting the release of nitric oxide (NO) from the cells into the culture supernatant and the expression of the TNF-α gene. Results in Fig. 2A show that LPS, a known inflammatory stimulator for macrophages^12^, strongly stimulates NO production in the RAW cells. In addition, the soluble β-glucan could not trigger NO production, while an equal amount of β-glucan on the beads could activate the release of NO from the cells in a dose-dependent manner. To rule out the possibility of endotoxin contamination, polymyxin B, an inhibitor of bacterial endotoxin, was applied to the experiment. The results show that while polymyxin B abolished LPS-induced NO production in the cells, it did not affect the NO production in LCB-stimulated cells (Fig. 2A). Additionally, the expression of TNF-α gene in the RAW 264.7 murine macrophages treated with LCB or LPS and in the presence or the absence of polymyxin B was analyzed. We find that LCB induces the expression of TNF-α approximately 2-fold better than LPS (Fig. 2B). Furthermore, this strong expression of TNF-α in the cells induced by the LCB is not affected by the presence of polymyxin B, which severely abolishes the TNF-α gene expression stimulated by LPS. Therefore, these results indicate that LCB is a potent inflammatory stimulator in RAW 264.7 murine macrophages and is not contaminated by bacterial endotoxin.

To compare the activity of LCB with the real fungal particles, NO production in RAW 264.7 murine macrophages treated with either LCB or heat-killed yeast particles was analyzed side by side. We find that all three particles (LCB, heat-killed *C. albicans*, and heat-killed *S. cerevisiae*) at the same concentration stimulate NO production in the cells in a dose-dependent fashion (Fig. 2C). However, the LCB induces the highest level of NO production in all concentrations. β-glucan on the actual fungal particles are partly hidden by proteins and other polysaccharides on their cell wall, whereas β-glucan (laminarin) on the LCB is not masked by other molecules. Additionally, chitin on the cell wall of the yeast may inhibit β-glucan recognition by Dectin-1^26^, contributing to a lower NO production in the cells. This observation is similar to Fig. 1 that compares the adipocyte activation by zymosan and heat-killed yeasts. Therefore, β-glucan on the LCB is highly accessible by the cells, explaining why LCB is superior to the real fungal particles in triggering an inflammatory response in the RAW 264.7 murine macrophages.

### Laminarin on beads is necessary and sufficient for eliciting an inflammatory response in RAW 264.7 murine macrophages

To illustrate the role of laminarin in eliciting an inflammatory response, we opted to digest laminarin with specific enzymes. Laminarin was predigested with either proteinase or glucanase enzymes prior to conjugation with the beads. We find that only the glucanase-digested but not the proteinase-digested laminarin on the beads lose their ability to promote NO production in the cells (Fig. 2D). These results could either suggest that laminarin on the beads is required for triggering the inflammatory response in the cells or that the digested laminarin could not be conjugated with the beads as efficient as the intact laminarin. However, we think the latter possibility is quite unlikely since the smaller the molecules, the less steric hindrance is produced. Therefore, the digested laminarin should easily be conjugated with the beads. Nonetheless, we also performed a digestion of LCB with glucanase enzymes (digestion after conjugation) and used the digested products to stimulate the cells. We find that the LCB digested with lyticase consistently shows a significant decrease in activating NO production when comparing with the undigested ones (Fig. 2E). Thus, these results suggest that laminarin on beads is necessary and sufficient for stimulating an inflammatory response in RAW 264.7 murine macrophages.

### Generation of LCB-induced RAW 264.7 murine macrophages medium (LCB-RM)

To mimic the effect of M1-polarized macrophages in an *in vitro* system, some researchers co-culture both macrophages and adipocytes together^27,28^. Alternatively, it was shown that both interferon-gamma (IFN-γ) and LPS can generate M1-polarized RAW 264.7 murine macrophages^29^, and the conditioned medium from RAW 264.7 cells treated with LPS stimulated inflammatory cytokine production in 3T3-L1 adipocytes^30^. Like LPS, *C. albicans* and *S. cerevisiae* were shown to differentially induce TNF-α production and release from macrophages^31^. However, the conditioned medium from actual fungal or fungal-like particle-treated RAW 264.7 murine macrophages has not been tested with adipocytes. Therefore, in addition to LCB, we explored the ability of this conditioned medium, namely LCB-treated conditioned medium from RAW 264.7 murine macrophages (LCB-RM), to elicit an inflammatory response in 3T3-L1 cells. The strategy of generating LCB-RM and the scope of the study is depicted in Fig. S2.

### LCB and LCB-RM induce an inflammatory response in differentiating adipocytes

**Figure 3:**
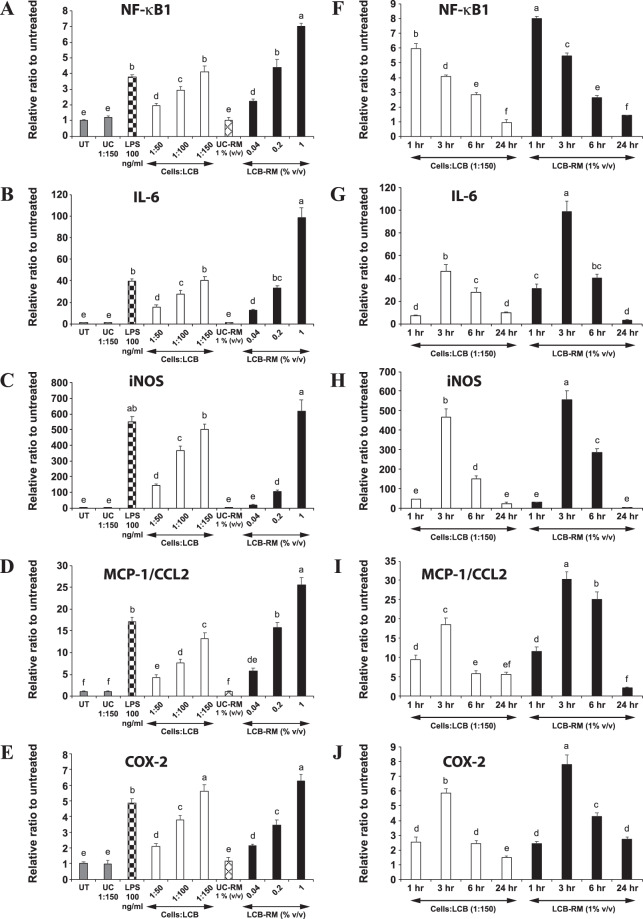
LCB or LCB-RM elicits an inflammatory response in differentiating 3T3-L1 adipocytes in dose- and time-dependent manners. (**A-E**) show the qPCR results of NF-κB1, IL-6, iNOS, MCP-1/CCL2, and COX-2 gene expression, respectively, in differentiating 3T3-L1 adipocytes treated with 1:50–1:150 cells:LCB ratios or with 0.04–1% (v/v) LCB-RM for 3 hours. LPS at a 100 ng/mL concentration was used as a positive control. UT, UC and UC-RM are abbreviated for untreated, unconjugated beads and unconjugated bead-treated conditioned medium from RAW 264.7 macrophages (UC-RM) respectively. (**F-J)** show the kinetic expression of NF-κB1, IL-6, iNOS, MCP-1/CCL2, and COX-2 genes, respectively, in differentiating cells treated with 1:150 cells:LCB ratio or with 1% LCB-RM (v/v) for 1–24 hours.

The experiments in the context of RAW 264.7 cells confirmed that our LCB is functional. Then, we proceeded to the experiment in the context of the fat cells, using LCB to test our hypothesis that the immobilized β-glucans on the fungal cell wall stimulate an inflammatory response in adipocytes. We performed the experiment in the context of both differentiating and differentiated 3T3-L1 adipocytes. For the experiments with adipocytes, the maximum amount of LCB that can be added to the cells is 1:150 cells:LCB ratio, which does not cause toxicity to adipocytes (Fig. S3A-B). Next, we examined whether LCB could stimulate expression of genes involved in the inflammation in differentiating adipocytes. Because the nuclear factor NF-kappa-B p105 subunit (NF-κB1) protein is one of the subunits in the canonical/classical NF-κB complex, which induces the expression of many genes, including inflammatory genes^16^, we analyzed the expression of NF-κB1. As for the dose-dependent analysis, differentiating adipocytes were treated with an increasing concentration of LCB for 3 hours. We find that the more LCB that is added, the greater the expression of NF-κB1 compared to the untreated cells or cells treated with uncoated beads (Fig. 3A). Next, we checked the expression of other inflammatory genes, which are IL-6, iNOS, MCP-1/CCL2, and COX-2. Consistent with NF-κB1 expression, the expression of those genes significantly increases as the amount of LCB increases (Fig. 3B-E). Additionally, LPS, which serves as a positive control, stimulates the expression of those genes (Fig. 3A–E). Together, these data demonstrate that LCB induces an inflammatory response in differentiating adipocytes in a dose-dependent manner.

In addition to LCB, we analyzed the effect of LCB-RM on the viability of the cells and whether it could stimulate an inflammatory response in differentiating adipocytes. We find that the LCB-RM at 1–4% v/v concentrations do not affect the viability of differentiating 3T3-L1 adipocytes (Fig. S3A–B). The analysis of gene expression reveals that LCB-RM induces the expression of those inflammatory genes in a dose-dependent fashion (Fig. 3A–E). Additionally, we observe that except for COX-2, the expression levels of NF-κB1, IL-6, iNOS, and MCP-1/CCL2 induced by LCB-RM at 1% are significantly higher than levels that are stimulated by LCB at the ratio of 1:150 cells:LCB (Fig. 3A–E).

Besides the dose-dependent effect, the kinetic expression of those inflammatory genes in LCB- and LCB-RM-treated differentiating adipocytes was investigated. For the NF-κB1 gene, we find that both LCB and LCB-RM stimulate the maximal expression of the gene during the first hour of treatment, and then the expression gradually declines from 3 to 24 hours of treatment (Fig. 3F). Unlike NF-κB1, the expressions of IL-6, iNOS, MCP-1/CCL2, and COX-2 reach the maximum at 3 hours and decline during 6 to 24 hours of incubation (Fig. 3G–J). The discrepancy in the kinetic expression patterns between NF-κB1 and the other genes is likely due to the fact that the NF-κB complex controls the expression of other genes^16^. In addition, the gradual decline in the gene expression of IL-6, iNOS, MCP-1/CCL2, and COX-2 after 3 hours of treatment suggests that the inflammatory inducing effect of LCB and LCB-RM happens transiently. Taken together, these data suggest that both LCB and LCB-RM stimulate an inflammatory response in differentiating adipocytes in a time-dependent manner.

### LCB and LCB-RM induce an inflammatory response in differentiated adipocytes

**Figure 4:**
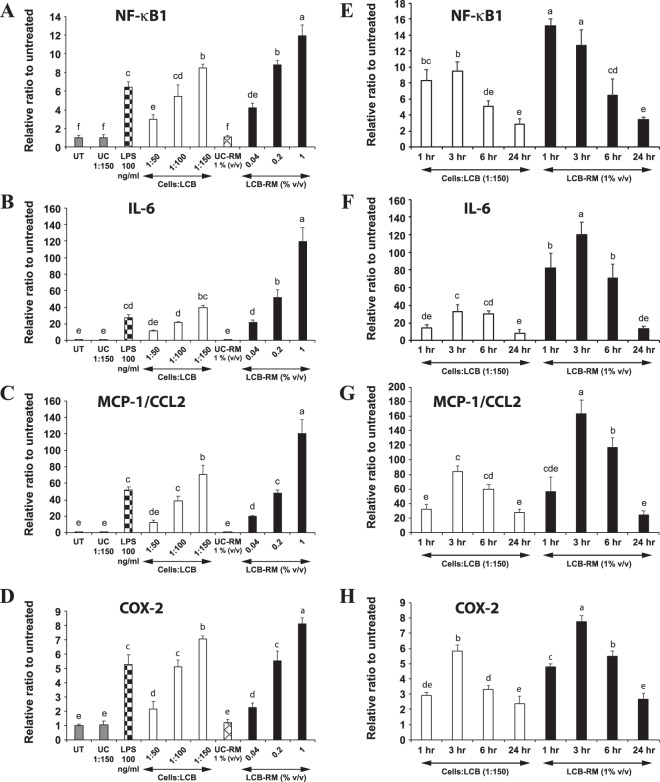
LCB or LCB-RM elicits an inflammatory response in differentiated 3T3-L1 adipocytes in dose- and time-dependent manners. (**A-D**) show the qPCR results of NF-κB1, IL-6, MCP-1/CCL2, and COX-2 gene expression, respectively, in differentiated 3T3-L1 adipocytes treated with 1:50–1:150 cells:LCB ratios or with 0.04–1% (v/v) LCB-RM for 3 hours. LPS at 100 ng/mL concentration was used as a positive control. UT, UC and UC-RM are abbreviated for untreated, unconjugated beads and unconjugated bead-treated conditioned medium from RAW 264.7 macrophages (UC-RM) respectively. (**E-H)** show the kinetic expression of NF-κB1, IL-6, MCP-1/CCL2, and COX-2 genes, respectively, in differentiated cells treated with 1:150 cells:LCB ratio or with 1% LCB-RM (v/v) for 1–24 hours.

Dose- and time-dependent effects of both LCB and LCB-RM in differentiated adipocytes were also investigated. We find that the expression of NF-κB1, IL-6, MCP-1/CCL2, and COX-2 genes are elevated as the increasing amount of LCB or LCB-RM was added to the cells, suggesting a dose-dependent effect of both stimulators (Fig. 4A–D). Like the differentiating adipocytes, LCB-RM at 1% (v/v) concentration appears to be a better inflammatory stimulator for the differentiated cells than LCB at 1:150 cells:LCB ratio (Fig. 4A–D). As for the kinetic effect, LCB-RM induces the expression of IL-6, MCP-1/CCL2 and COX-2 the same pattern as it does to the differentiating cells (Fig. 4E–H). NF-κB1 gene expression in the differentiated cells induced by LCB-RM shares a similar pattern as it is in the differentiating cells, except that the expression at 1 and 3 hours are statistically insignificantly different (Fig. 4E). Unlike LCB-RM, LCB produces differential effects on the kinetic expression of those genes. The kinetic expression of COX-2 genes induced by LCB reaches the maximum at 3 hours and declines onward (Fig. 4H). However, the expression level of NF-κB1 induced by LCB during the first 3 hours is statistically indifferent, whereas the IL-6 and MCP-1/CCL2 gene expressions activated by LCB during 3 to 6 hours are statistically identical (Fig. 4E–G).

From the gene expression data in Figs. 3 and 4, we calculated the ratio between the level of gene expression in differentiated adipocytes to differentiating adipocytes (D:D ratio) induced by LCB or LCB-RM (Fig. S4). We find that in most cases, dose and time treatments with LCB or LCB-RM do not result in significant changes in the D:D ratio. Secondly, the D:D ratios of NF-κB1 and MCP-1/CCL2 in most cases are above 1, suggesting a greater gene expression in differentiated adipocytes. In contrast, the ratios of IL-6 and COX-2 (e.g. from the increasing amount of LCB for 3 hours) are approximately 1 or less, suggesting a greater gene expression in differentiating adipocytes. Lastly, the D:D ratios of MCP-1/CCL2 gene expression induced by LCB or LCB-RM are remarkably high when compared with other genes, suggesting a greater gene expression in differentiated adipocytes.

A high expression of MCP-1/CCL2 gene in differentiated adipocytes is consistent with the previous finding that this protein contributes to macrophage infiltration into the adipose tissue^3^. Therefore, these results suggest that the differentiated cells respond to those stimulators better than the differentiating cells.

Although the inflammatory-inducing effect of fungal particles or fungal-like particles on 3T3-L1 adipocytes has not previously been illustrated, the effect of LPS on inflammatory gene expression in 3T3-L1 adipocytes has been shown. Thus, we compare our inflammatory gene expression data with a previous study by Chirumbolo and colleagues that analyzed dose and time effects of LPS on inflammatory cytokine production, including IL-6, TNF-α, macrophage inflammatory protein 1-alpha (MIP-1α/CCL3), MIP-1β/CCL4, C-X-C motif chemokine 12 (CXCL12), and IL-10 in 3T3-L1 adipocytes^32^. We attempted but could not detect the expression of TNF-α and IL-10. Interestingly, the study by Chirumbolo and colleagues shows an increasing trend of IL-6 gene expression due to the increasing concentration and prolong incubation with LPS^32^. The difference in the kinetic expression of IL-6 in this and their studies probably reflects the differential stimulation between LPS and our inflammatory stimuli (LCB and LCB-RM). However, the direct comparison cannot be made because LPS only serves as a positive control and is not the focus of this study.

### IκBα negatively regulates LCB- and LCB-RM-induced inflammatory response in both differentiating and differentiated 3T3-L1 adipocytes

**Figure 5:**
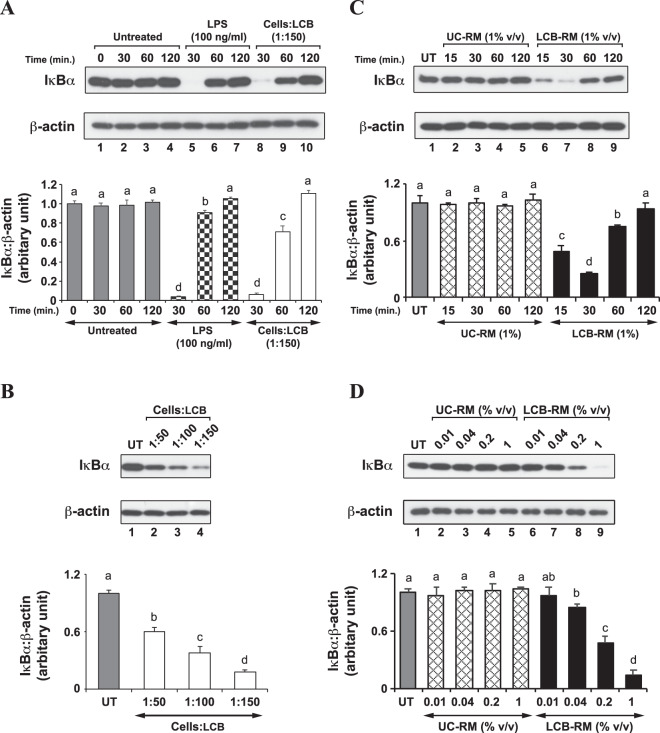
LCB or LCB-RM promotes dose- and time-dependent degradation of IκBα in differentiating 3T3-L1 adipocytes. (**A**), a representative Western blot data set shows kinetic degradation of IκBα protein. Differentiating 3T3-L1 adipocytes were treated with LCB at the ratio of 1:150 cells:LCB, and the cells were collected at 30, 60 and 120 minutes for Western blot analysis using anti-IκBα and anti-β-actin antibodies. LPS at 100 ng/mL concentration was used as a positive control. The IκBα:β-actin protein intensity ratio at each time point was calculated and expressed as the relative ratio with respect to the value from the untreated at zero minute, as shown in the chart. UT is abbreviated for untreated. (**B)**, a representative Western blot data set shows dose-dependent effect of LCB on IκBα degradation. Differentiating 3T3-L1 adipocytes treated with an increasing amount of LCB for 30 minutes were collected for Western blot analysis using anti-IκBα and anti-β-actin antibodies. The intensity ratio of IκBα:β-actin proteins were calculated and shown in the chart as described in (**A)**. (**C**,**D**) are like (**A**,**B**), respectively, except that 1% v/v LCB-RM was used to induce IκBα protein degradation. UC and UC-RM are abbreviated for unconjugated beads and unconjugated bead-treated conditioned medium from RAW 264.7 macrophages (UC-RM).

Previously, a study showed that LPS inhibited adipogenesis by causing IκBα degradation^14^. These results led us to speculate whether LCB and LCB-RM would promote IκBα protein degradation in adipocytes. Therefore, we analyzed the IκBα protein by mean of Western blotting, using anti-IκBα antibodies in the context of both differentiating and differentiated 3T3-L1 adipocytes. In the differentiating cells, we find that the IκBα protein is severely degraded at 30 minutes but resumes the normal level within 120 minutes upon treatment with either LPS or LCB, while the IκBα protein is stable throughout the time period in the untreated set (Fig. 5A). In addition, by treating the differentiating adipocytes with LCB in an increasing dose for 30 minutes, the IκBα protein level decreases as the concentration of the LCB increases (Fig. 5B). Consistently, LCB-RM induces the degradation of IκBα protein in the same manner as the LCB (Fig. 5C,D). Therefore, these results indicate that the IκBα protein involves in both LCB- and LCB-RM-induced inflammatory response in differentiating 3T3-L1 adipocytes.

**Figure 6:**
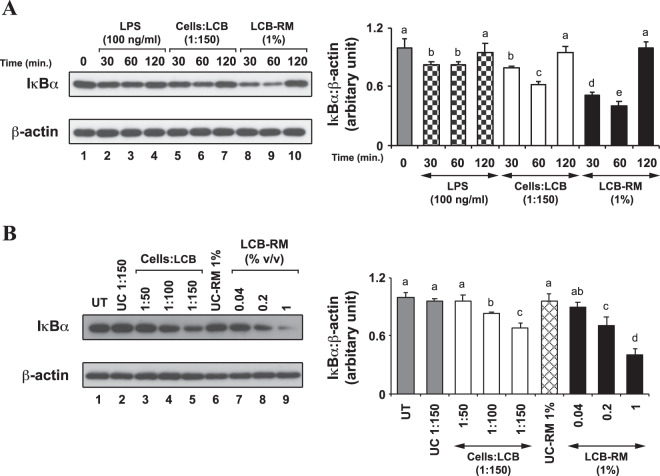
LCB or LCB-RM promotes dose- and time-dependent degradation of IκBα in differentiated 3T3-L1 adipocytes. **(A)**, a representative Western blot data set shows the kinetic degradation of IκBα protein. Differentiated 3T3-L1 adipocytes were treated with LCB at the ratio of 1:150 cells:LCB or with 1% LCB-RM, and the cells were collected at 30, 60 and 120 minutes for Western blot analysis using anti-IκBα and anti-β-actin antibodies. LPS at 100 ng/mL concentration was used as a positive control. The IκBα:β-actin protein intensity ratio at each time point was calculated and expressed as the relative ratio in respect to the value from the untreated at zero minute, as shown in the chart. (**B)**, a representative Western blot data set shows dose-dependent effect of LCB or LCB-RM on IκBα degradation. Differentiated 3T3-L1 adipocytes treated with an increasing amount of LCB or LCB-RM for 30 minutes were collected for Western blot analysis using anti-IκBα and anti-β-actin antibodies. The intensity ratio of IκBα:β-actin proteins were calculated and shown in the chart as described in (**A)**. UC and UC-RM are abbreviated for unconjugated beads and unconjugated bead-treated conditioned medium from RAW 264.7 macrophages (UC-RM).

In addition, the degradation of IκBα protein in the differentiated cells was analyzed. Unlike the differentiating cells, the degradation of IκBα protein in the differentiated cell stimulated by LPS, LCB and LCB-RM is not nearly as complete as that in the differentiating cells, although LCB-RM appears to cause more IκBα protein degradation than LCB at a 60-minute time point (Fig. 6A). We cannot explain the discrepancy between the maximum time point for IκBα protein degradation in differentiating and differentiated cells. Perhaps this reflects differential sensitivity in the two forms of the cells. Moreover, the maximum degradation of IκBα protein in the cells is at 60 minutes (Fig. 6A). Also, the LCB-RM at 1% (v/v) concentration seems to be a better inducer of IκBα degradation than the LCB at 1:150 cells:LCB ratio (Fig. 6B). This observation is consistent with the results in Fig. 4A–D, in which 1% (v/v) concentration of LCB-RM induces a greater level of gene expression than the LCB at the highest cells:LCB ratio.

The role of IκBα protein in an inflammatory response triggered by both LCB and LCB-RM is further investigated. It has been known that the inhibitor of nuclear factor kappa-B kinase subunit beta (IKKβ) protein phosphorylates and subsequently causes proteasomal degradation of IκBα protein^33^. Therefore, the inhibition of either IKKβ or proteasome complex would stabilize the IκBα protein. To test this hypothesis, we utilized SC-514 (an inhibitor of IKKβ) and MG132 (a proteasome inhibitor) to treat the differentiating 3T3-L1 adipocytes prior to an incubation with LCB or LCB-RM. The experiment was only performed in the context of differentiating cells because the degradation of IκBα is more pronounced in the differentiating cells than the differentiated cells.

**Figure 7:**
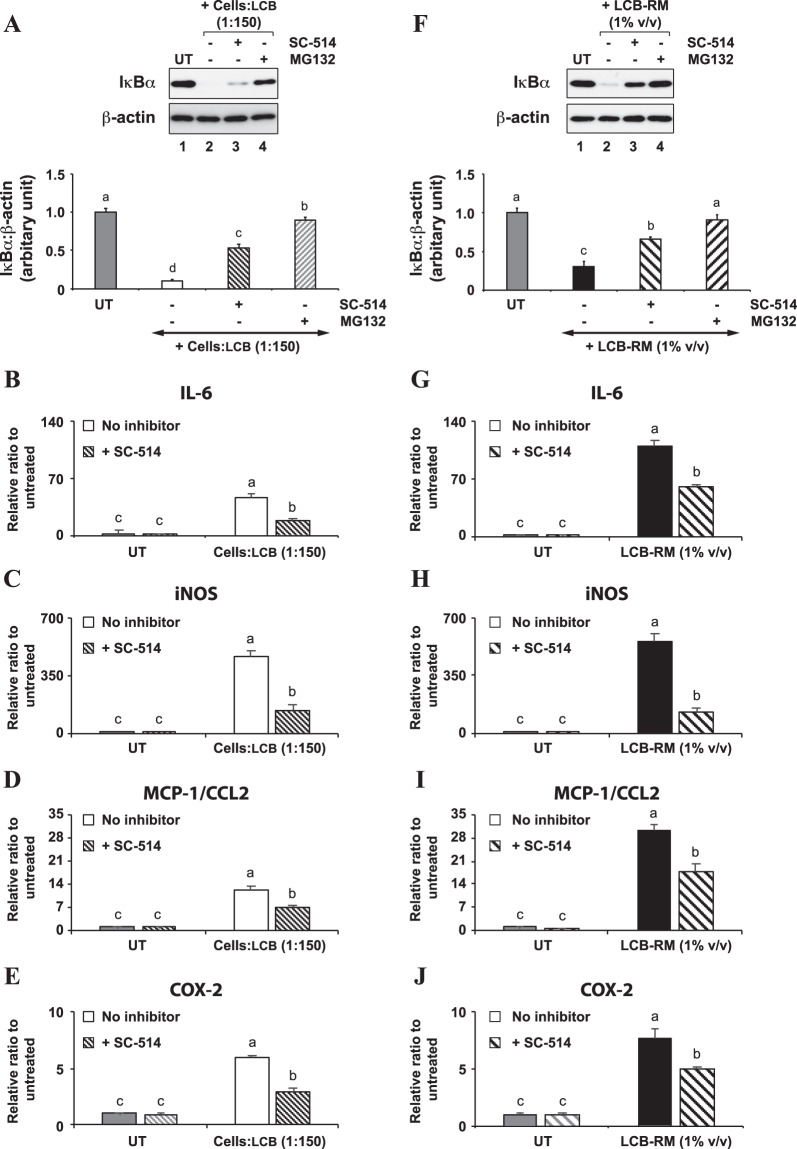
IKKβ promotes proteasomal degradation of IκBα, which negatively regulates the expression of inflammatory genes. (**A**), a representative Western blot data set shows the effects of inhibitors on IκBα protein degradation induced by LCB. Differentiating 3T3-L1 adipocytes treated for 30 minutes with LCB (1:150 cells:LCB) and with or without 50 μM SC-514 (IKKβ inhibitor) or 20 μM MG132 (proteasome inhibitor) were collected for Western blot analysis using anti-IκBα and anti-β-actin antibodies. The IκBα:β-actin protein intensity ratio at each treatment was calculated and expressed as the relative ratio in respect to the value from the UT (untreated), as shown in the chart. (**B**–**E)**, qPCR results show the effect of SC-514 inhibitor on inflammatory gene expression (IL-6, iNOS, MCP-1/CCL2, and COX-2) induced by LCB. Differentiating 3T3-L1 adipocytes were treated for 3 hours with LCB (1:150 cells:LCB) in the presence or the absence of 50 μM SC-514 inhibitor. (**F**–**J)** are like (**A**–**E)**, respectively, except that 1% v/v LCB-RM was used to induce IκBα protein degradation.

We find that pretreatment of adipocytes with either SC-514 or MG132 confers a stability of IκBα protein in the differentiating adipocytes treated with either LCB or LCB-RM (Fig. 7A,F). In addition, we tested the requirement of IκBα on the expression of inflammatory genes. Because the IκBα protein blocks nuclear translocation of NF-κB complex^16^, inhibition of IκBα degradation should downregulate the expression of NF-κB target genes, which include inflammatory genes. To test this idea, we analyzed the expression of inflammatory genes in differentiating adipocytes treated with SC-514. The inhibitor significantly suppresses the expression of IL-6, iNOS, MCP-1/CCL2, and COX-2 genes induced by either LCB or LCB-RM (Fig. 7B–E,G–J). Evidently, the fall in IκBα protein level during the first 30 minutes (Fig. 5A,C) causes a significant upregulation of IL-6, iNOS, MCP-1/CCL2, and COX-2 genes observed at 3 hours in Fig. 3G–J. Conversely, the rise in the IκBα protein level after 30 minutes (Fig. 5A,C) results in a gradual decrease in those gene expression after 3 hours (Fig. 3G–J). Together, these results suggest a negative role of IκBα protein on the inflammatory gene expression in differentiating 3T3-L1 adipocytes triggered by either LCB or LCB-RM. In other words, the canonical NF-κB pathway mediates the expression of those inflammatory genes in differentiating adipocytes driven by either LCB or LCB-RM. As explained above, this conclusion is also true for the differentiated cells.

Given the involvement of the IκBα protein, one would predict that the more IκBα degraded, the greater the expression of those inflammatory genes. Thus, the levels of gene expression in differentiating cells induced by either LCB or LCB-RM should be greater than that in the differentiated cells. However, this prediction is inconsistent with the observation in Fig. S4, in which the expression of MCP-1/CCL2 in differentiated adipocytes is higher than that in the differentiating cells. The gene expression could not be judged by the reduction of a negative regulator (IκBα) alone. The contribution of a positive regulator (NF-κB1), as part of the NF-κB complex, which is required for the expression of inflammatory genes, should also be accounted for. Because the expression of NF-κB1 in differentiated adipocytes is higher than that in differentiating adipocytes (D:D ratio is about 2, Fig. S4), a high expression level of inflammatory genes in the differentiated cells is observed, especially that of MCP-1/CCL2 gene.

## Discussion

**Figure 8:**
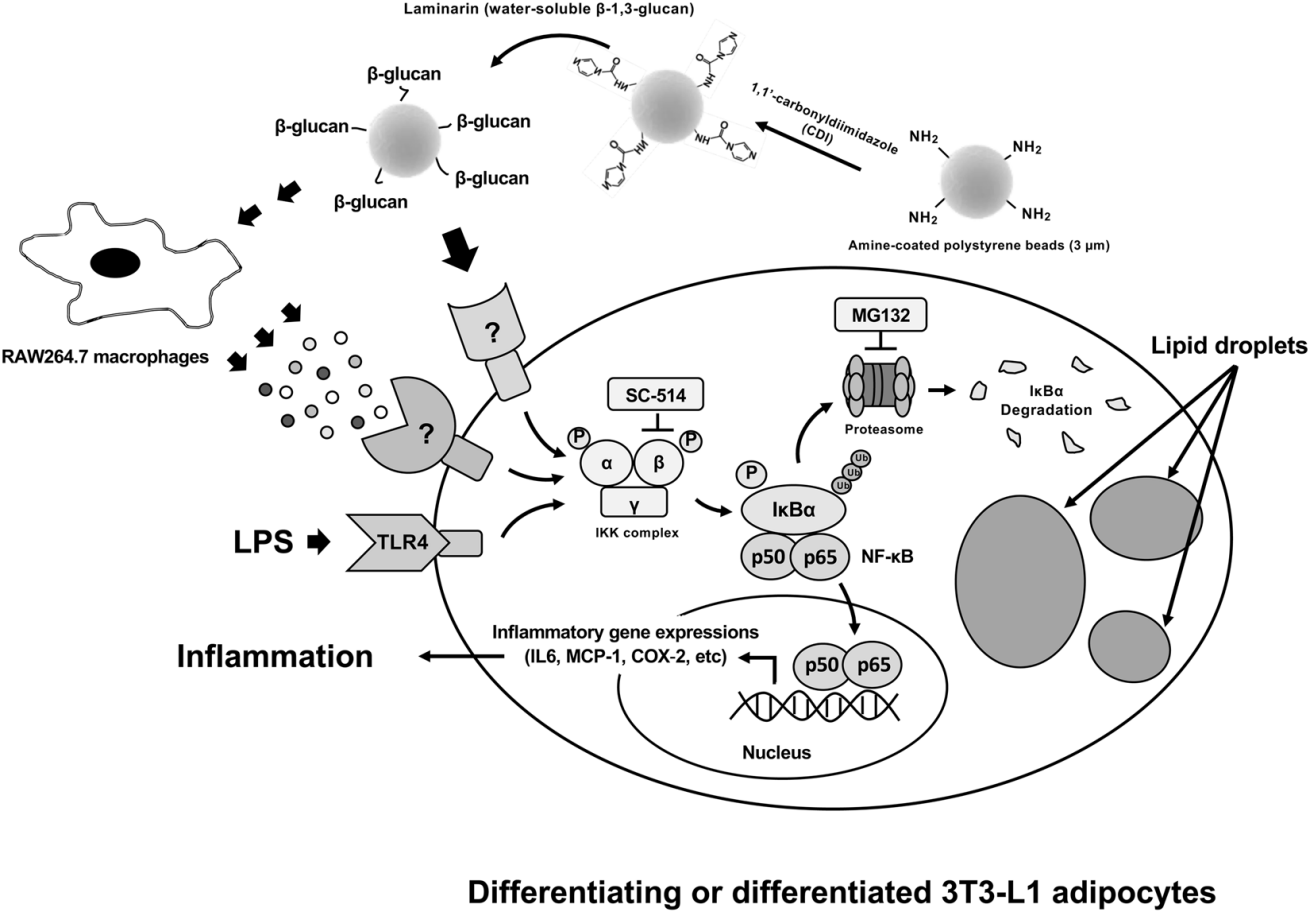
A proposed mechanism of an inflammatory response in differentiating or differentiated 3T3-L1 adipocytes triggered by LPS, LCB or LCB-RM. Fungal-like particles namely LCB was created from laminarin and polystyrene beads. LCB binds to a specific, unidentified surface receptor on adipocytes. The receptor subsequently triggers a downstream signaling pathway, leading to the activation of the IKK complex, which consists of IKKα, IKKβ and IKKγ/NEMO subunits. The activation of the IKK complex can be inhibited by SC-514, a specific inhibitor of the IKKβ subunit. The activated IKK complex phosphorylates the IκBα protein, which forms a complex with NF-κB, a heterodimer of p50 (NF-κB1) and p65 (RelA) subunits. Phosphorylated IκBα is targeted for destruction by the 26S proteasome complex, causing the release and nuclear translocation of NF-κB. NF-κB promotes transcription and production of inflammatory cytokines, leading to inflammation of adipose tissue.

In this study, we show that heat-killed yeast particles stimulate an inflammatory response in adipocytes. Using fungal-like particles, namely LCB, we further show that LCB elicits an inflammatory response in both differentiating and differentiated 3T3-L1 adipocytes through the canonical NF-κB pathway, implying that β-glucan on fungal particles is sufficient to trigger an inflammatory response in adipocytes. Additionally, the conditioned medium from LCB-treated macrophages or LCB-RM stimulates an inflammatory response in adipocytes through the same signaling pathway. The mechanistic effect of LCB and LCB-RM on inflammation in differentiating adipocyte is depicted in Fig. 8.

Although we demonstrate that the LCB-RM induces an inflammatory response, we did not experimentally identify the active molecules in the LCB-RM. However, some potential components in the conditioned medium that activate inflammation in adipocytes were suggested. For example, mice injected with TNF-α showed a significant increase in IL-6 proteins in the serum^34^. Furthermore, 3T3-L1 adipocyte treated with TNF-α showed a time-dependent induction of IL-6 gene expression^35^. Consistently, we show that TNF-α stimulates the IL-6 and MCP-1/CCL2 gene expressions (Fig. S5). In addition, a previous study showed that NF-κB complex mediates the lipolysis in human adipocytes induced by TNF-α^36^. Interleukin-1 beta (IL-1β) is also another candidate. As shown by Gao and colleagues, IL-1β inhibited expression of many genes in insulin signaling and stimulated expression of inflammatory cytokines, including IL-6 and MCP-1/CCL2 in human adipocytes^37^. Also, conditioned medium prepared from both THP-1 macrophages cell line and monocyte-derived macrophages mimicked the IL-1β treatment. Additionally, blocking IL-1β activity using the antibodies that bind to IL-1β, preventing the release of IL-1β from macrophages or blocking the IL-1 receptor on human adipocytes, reversed the effect of that conditioned medium^37^. Together, those findings suggest that either TNF-α or IL-1β or both proteins are the potential inflammatory mediators in the LCB-RM.

The effect of macrophage conditioned medium is not limited to the induction of inflammation as it was also shown to inhibit adipogenesis^38–40^, suggesting a possible adipogenesis inhibition activity from the LCB-RM. Like the conditioned medium, a previous study showed that particulate β-glucan from yeast inhibited adipogenesis in differentiating adipocytes^41^. This study suggests that fungal particles may inhibit adipogenic differentiation. Because the LCB mimics fungal particles, we predict that our LCB may inhibit adipogenesis.

In order for β-glucan to trigger an inflammatory effect, it must be in a particulate or insoluble form. Results from our study show that soluble β-glucan is not able to induce an inflammatory response in RAW 264.7 murine macrophages, and that is likely true with 3T3-L1 adipocytes. However, the effect of soluble β-glucan in the context of the whole body is different. A recent study showed that oral administration of soluble yeast β-glucan inhibited inflammatory gene expression in visceral adipose tissue (VAT), improved the gut microbiota, and reduced inflammation in the intestine of the leptin mutant mice (*ob*/*ob* mice), which develop obesity and diabetes, similar to individuals with type-II diabetes^42^. Similarly, other soluble forms of β-glucan from bacteria, plant and mushroom were shown to improve glucose tolerance, reduce insulin resistance, and induce anti-inflammation and anti-obesity responses in high-fat diet fed mice^43–46^. Therefore, soluble β-glucan promotes positive effects on the body. Consistently, it was shown that soluble laminarin prevented body weight gain and improved glucose tolerance in mice fed with a high-fat diet^47^. Yet, the authors did not show an anti-inflammation of laminarin *in vivo* and we did not show whether soluble laminarin would inhibit the activity of LCB on adipocytes. Nonetheless, we would predict that soluble laminarin exhibits an anti-inflammation *in vivo*.

While both LCB and LCB-RM induce a strong response in adipocytes, the heat-killed yeast particles stimulate a weak inflammatory response. The expression of IL-6 and MCP-1 gene expression in adipocytes induced by the heat-killed yeast is significantly different from the untreated control but very low when compared with LPS or zymosan. However, the same heat-killed yeast induced a significant amount of NO in the RAW 264.7 murine macrophages, albeit lower than that induced by LCB. These results suggest that there is a specific β-glucan receptor on the cell surface of adipocytes. In addition, macrophages are more sensitive to fungal particles than adipocytes. In other words, adipose tissue is not likely a major target of fungal pathogens because neither fungal infection nor fungal-related diseases in adipocytes have been reported. Adipocytes may be less sensitive to fungal particles than macrophages due to receptors on the cell surface. Adipocytes may produce a lesser amount of the receptors than macrophages, conferring adipocytes less accessibility and sensitivity to the heat-killed yeast than macrophages. Another possibility is that adipocytes may recognize β-glucan through a different receptor from macrophages. Previous studies have suggested TLR2 as a possible candidate receptor of β-glucan on the cell surface^15,18^. However, it is not clear if TLR2 is the specific β-glucan receptor because it is not normally expressed in the cells^18^. Interestingly, a recent study suggests the role of CD36, a scavenger receptor, as a β-glucan receptor in both macrophages and 3T3-L1 adipocytes^48^. Thus, future investigation is required to identify the specific β-glucan receptor on adipocytes.

## Materials and methods

### Conjugation of polysaccharides on polystyrene beads

The method for conjugating laminarin with polystyrene beads was slightly modified from the previously published protocol by Tam and colleagues^20^. Briefly, 3 μm aliphatic amide latex beads (Thermo Scientific) were used for polysaccharide conjugation. Briefly, 300 μL of the resuspended beads were washed with dimethylsulfoxide (DMSO, Sigma-Aldrich) for 3 times using centrifugal filters PTFE membrane with 0.45 μm pore size (Ultrafree brand, Millipore). After resuspending the beads in 1 mL anhydrous DMSO, 1,1′-carbonyldiimidazole (CDI, Sigma-Aldrich) was added to a final concentration of 0.5 M. The beads were transferred to a clean Eppendorf tube and shaken at room temperature for 1 hour before washing 3 times with anhydrous DMSO using a centrifugal filter. The beads were resuspended in 10 mg/mL laminarin to allow a coupling reaction with CDI activated beads. After 12–16 hours of incubation at room temperature, the conjugated beads were washed with 1xPBS 3 times using a centrifugal filter. The presence of β-glucan on the beads was tested with the phenol-sulfuric acid assay as previously described^49^.

### Heat-killed yeast particles

Preparation of the heat-killed yeast particles was performed as described previously^50^. In brief, a single colony of either *C. albicans* or *S. cerevisiae* was inoculated in YPD medium (1% yeast extract, 2% peptone and 2% glucose) and incubated in an orbital shaker at 28 °C, 200 rpm for 12–16 hours. The culture was then diluted 1:100 in fresh YPD and allowed to grow for 4 hours. Cells were harvested and washed three times with 1xPBS. The heat-killed yeast particles were prepared by resuspending the cell pellet in 1xPBS and incubated at 65 °C for 30 minutes. The density of yeast particles was determined using the Bright-LineTM Hemacytometer (Sigma-Aldrich).

### Digestion of laminarin with enzymes

In this study, either laminarin or LCB was subjected to enzymatic digestion. The digestion of soluble laminarin was performed with either glucanases or proteinases. To digest soluble laminarin with glucanases, laminarin was dissolved in 0.2 M sodium acetate buffer pH 5.0 and 250 U/mL lyticase (Sigma-Aldrich) was added. After 1 hour of incubation at 37 °C, the sample was subsequently digested with glucanase enzyme mix (Megazyme, Ireland), which contains 1 U/mL exo-1,3-β-glucanase and 0.2 U/mL β-glucosidase, final concentrations, at 40 °C for 12–16 hours. To digest the protein, laminarin was dissolved in the HC buffer (10 mM HEPES pH 7.4 and 1 mM CaCl_2_) and 100 U/mL proteinase K was added. The digestion was performed at room temperature for 12–16 hours before the conjugation reaction. BSA was used as a positive control for proteinase K digestion.

The digestion of LCB was performed similarly. The LCB was subjected to digestion with the same concentration of lyticase but without further digestion with the enzyme mix from Megazyme. The digestion of LCB with proteinase K was performed exactly as described above. Digested LCB was washed with 1x phosphate buffered saline (PBS) before testing with RAW 264.7 murine macrophages.

### Cell cultures

RAW 264.7 murine macrophages and 3T3-L1 adipocytes were purchased from the American Type Culture Collection (ATCC) and cultured in complete Dulbecco’s modified Eagle’s medium (cDMEM, Sigma-Aldrich) containing 10% fetal bovine serum (FBS), 1% Penn-Strep (100 U/mL Penicillin and 100 μg/mL Streptomycin), L-glutamine, and sodium pyruvate at 37 °C and 5% CO_2_.

### RAW 264.7 murine macrophages stimulation with LCB

To analyze the effects of LCB on RAW 264.7 murine macrophages, the Griess assay was performed. Briefly, RAW 264.7 murine macrophages were seeded in a 96-well plate at the density of 1 × 10^5^ cells/well. After 24-hour seeding, cells were washed once with 1xPBS, and fresh cDMEM medium without phenol-red supplemented with heat-killed yeasts, soluble laminarin, LCB, or uncoated beads at a desired concentration was added. LPS at a 10 ng/mL final concentration was used as a positive control. After incubating for 24 hours, the medium was collected for the Griess assay, which is described previously^51^, whereas the cells were used for the viability assay using 3-[4,5-dimethylthiazole-2-yl]-2,5-diphenyltetrazolium bromide (MTT) reagent^52^.

For quantitative PCR (qPCR), the assay was performed in a similar manner, except that the cells were seeded in a 48-well plate at a density of 2 × 10^5^ cells/well. After the 24-hour seeding, the medium was replaced with fresh medium containing LPS, LCB or uncoated beads without washing the cells. Cells were treated for 6 hours before being harvested for qPCR assay.

To verify whether the conjugated beads were contaminated with endotoxin, polymyxin B at a 10 μg/mL final concentration was added together with LPS, LCB or uncoated beads. The Griess and qPCR assays were conducted as described above.

### Production of LCB-treated RAW 264.7 cell-conditioned medium (LCB-RM)

RAW 264.7 murine macrophages were treated with LCB as described above. After 24-hour incubation, the cell-free supernatant was collected and filtered through a 0.2 μM filter. The resulting LCB-RM was used to treat differentiating and differentiated 3T3-L1 adipocytes at a desired concentration (e.g. 1% v/v).

### Differentiation of 3T3-L1 adipocytes

3T3-L1 fibroblasts can be differentiated into adipocytes as previously described^53^. On the first day, 3T3-L1 fibroblasts at 2 × 10^4^ cells were seeded in a 48-well plate. The medium was replenished on the third day when the cells reached 100% confluency. On the 5^th^ day, the medium was changed to the D/A medium (10% FBS, 2 mM penicillin–streptomycin, 1 μM dexamethasone, 0.5 mM 3-isobutyl-1-methylxanthine (IBMX), 10 μg/mL insulin, and DMEM media), which initiates the differentiation process. Then, on the 8^th^ day, the medium was changed to the D/M medium (10% FBS, 2 mM penicillin–streptomycin, 10 μg/mL insulin, and DMEM media). The medium was changed again on the 10^th^ day to the fresh D/M medium. Differentiated cells were ready to be assayed on the 12^th^ day. Fully differentiated cells contain lipid-droplet accumulation in the cytosol, which was easily observed under a light microscope.

### Treating differentiating or differentiated adipocytes with inflammatory stimulators

To analyze the effect of inflammatory stimulators (e.g. LPS, LCB or LCB-RM) on differentiating cells, each stimulator at a desired concentration (e.g. 1:150 cells:LCB or 1% LCB-RM) was mixed with the D/A medium and added to the cells. For the time-dependent effect, the incubation time (1–24 hours) was performed before collecting the cells. For the dose-dependent effect, cells were incubated with the increasing amount of each stimulator for 3 hours or 30 minutes before being harvested for qPCR or Western blotting, respectively. Likewise, the effect of inflammatory stimulators (e.g. heat-killed yeast, zymosan, LPS, LCB, or LCB-RM) on differentiated cells was accessed as above but using the differentiated cells.

### Total RNA extraction and complementary DNA (cDNA) synthesis

Total RNA extraction experiment was performed using TRIZOL reagent (Invitrogen) as recommended by the manufacturer. After homogenizing the cell culture with TRIZOL, chloroform was added and mixed. The aqueous phase, which contains RNA, was precipitated with isopropanol and used for quantitation at A260 absorbance, gel electrophoresis and cDNA synthesis. To create cDNA, reverse transcriptase (Thermo Scientific) was used as recommended by the manufacturer.

### Quantitative PCR (qPCR)

qPCR was performed using a commercialized pre-mixed qPCR reagent that contains EvaGreen fluorescence dye (Solis BioDyne) on a real-time PCR machine (Aligent Technologies, Stratagene Mx3005P). The list of primers used in this study is shown in Table S1. Data were analyzed using the 2^−ΔΔCt^ method as described previously^54^.

### Western blotting assay

Western blotting was performed with cell lysates from treated adipocytes. Briefly, the treated cells were resuspended with ice-cold T150 buffer (50 mM Tris-Cl pH 7.4, 150 mM NaCl, 1 mM EDTA, 0.5% NP-40, 1 mM NaF, 1 mM Na_3_VO_4_, and 1x protease inhibitor cocktail from Roche) and left on ice for 30 minutes, with a vortex every 5 minutes. The sample was subsequently centrifuged at 13,200 rpm at 4 °C for 30 minutes. The soluble lysate was transferred to a clean tube and mixed 1:1 with 2x protein gel loading buffer (12.5 mM Bis-Tris pH 6.8, 20% glycerol, 4% SDS, 2.16 mM 2-mercaptoethanol, and 0.04% bromophenol blue). Proteins from the lysates were resolved with SDS-PAGE and transferred to a PVDF membrane. After rinsing with distilled water, the membrane was incubated for 5–10 minutes with 25 ml 1xTBST buffer, which contains 0.025 M Tris pH 8.0, 150 mM NaCl and 0.05% Tween 20. Then, the membrane was incubated in a blocking solution (5% milk in 1xTBST) for 30 minutes. Subsequently, the membrane was incubated for 12–16 hours at 4 °C with a desire primary antibody (e.g. anti-β-actin from Merck or anti-IκBα from ABCAM) diluted 1:10,000 with blocking solution. Following the 3 washes with 1xTBST for 5-minute each, the membrane was incubated for 1 hour at room temperature with HRP-conjugated goat-anti-rabbit antibodies (Merck), diluted 1:20,000 with blocking solution. The membrane was then washed again as described above. The signal from the secondary antibodies was revealed with chemiluminescence substrates. The membrane was exposed to an X-ray film (Pierce) at a desired exposure time. The X-ray film was developed with developing solutions purchased from Kodak.

### Assay with inhibitors

The inhibitors used in this study include SC-514 and MG-132. Briefly, after 30 minutes of incubation with the inhibitors (50 μM for SC-514 and 20 μM for MG132), the medium was removed. Then, fresh D/A medium supplemented with the inhibitor at the same concentration and LCB or LCB-RM at the desired amount was added to the cells. Cells were harvested for Western blotting or qPCR after 30 minutes or 3 hours of the treatment, respectively.

### Statistical analysis

All experiments were performed at least three times independently and each data set was performed in triplicate. The error bars represent standard deviations. Statistical differences were calculated using one-way ANOVA with Duncan’s multiple range tests (*p* < 0.05) by the statistical software R (version 3.6.2).

## Supplementary information

Supplementary information.

## Data Availability

All data generated or analyzed during this study are included in this published article (and its Supplementary Information files).
