# Supplementary material for: Fungal-like particles and macrophage-conditioned medium are inflammatory elicitors for 3T3-L1 adipocytes
Source: Sci Rep. 2020 Jun 10;10:9437. doi: 10.1038/s41598-020-66283-4 (PMC7287055; doi:10.1038/s41598-020-66283-4)
Supplement: Supplementary file 1 — Supplementary information. [file 41598_2020_66283_MOESM1_ESM.docx]

**Fungal-like particles and macrophage-conditioned medium are inflammatory elicitors for 3T3-L1 adipocytes**

Chanawee Jakkawanpitak^1^, Nongporn Hutadilok-Towatana^1^ and Decha Sermwittayawong^*1^

^1^*Department of Biochemistry, Faculty of Science, Prince of Songkla University, Hat Yai, 90110, Thailand*

*corresponding author: [decha.s@psu.ac.th](mailto:decha.s@psu.ac.th), tel: +66-82-5868247, ORCID ID: <https://orcid.org/0000-0001-8941-3642>

**Table S1**: Primers used in this study

| **Gene** | **Types** | **Primer sequences** |
| --- | --- | --- |
| mTNF-α | Forward | CGAGTGACAAGCCTGTAGCCC |
|  | Reverse | GTCTTTGAGATCCATGCCGTTGG |
| mIL-6 | Forward | GAGGATACCACTCCCAACAGACC |
|  | Reverse | CTGCAAGTGCATCATCGTTGTTC |
| mNF-κB | Forward | TGGCAGACGATGATCCCTACG |
|  | Reverse | CGGAATCGAAATCCCCTCTGTT |
| miNOS | Forward | TTGGAGCGAGTTGTGGATTGTC |
|  | Reverse | GCAGCCTCTTGTCTTTGACCCAG |
| mMCP-1 | Forward | CTCATTCACCAGCAAGATGATCC |
|  | Reverse | CCTTCTTGGGGTCAGCACAG |
| mCOX-2 | Forward | CCTCTGCGATGCTCTTCC |
|  | Reverse | TCACACTTATACTGGTCAAATCC |
| mActin | Forward | CCTGAGGCTCTTTTCCAGCC |
|  | Reverse | GCCAGAGCAGTAATCTCCTTCTG |

**Fig. S1: LCB up to 1:30 cell:beads ratio is not toxic to RAW 264.7 murine macrophages.** Cell viability of RAW 264.7 murine macrophages treated with either unconjugated beads (UC) or laminarin-coated beads (LCB) at 3 different ratios of cells:beads.

**Fig. S2: Strategy of the experiments**

**Fig. S3: Neither LCB nor LCB-RM is toxic to differentiating and fully differentiated adipocytes. A,** Differentiating 3T3-L1 adipocytes were treated with various concentrations of LCB and LCB-RM. UT, UC and UC-RM are negative control sets and abbreviated for untreated, unconjugated beads and unconjugated beads RAW 264.7 conditioned medium, respectively. All treatments are not toxic to the cells. **B,** The data are similar to A, except that fully differentiated 3T3-L1 adipocytes were used. IPR is abbreviated for isoproterenol.

**Fig. S4: Comparing the effects of either LCB or LCB-RM on fully differentiated and differentiating adipocytes**. The gene activation data from figures 2 and 3 were used to calculate the relative gene activation ratio between fully differentiated and differentiating adipocytes. Diff. is abbreviated for differentiating. **A** and **B** show relative gene expression ratios of the cells treated with 3 different ratios of cell:LCB for 3 hours or with 3 concentrations of LCB-RM, respectively. **C** and **D** show the kinetics of relative gene expression ratios of the cells treated with 1:150 cell:LCB ratio or 1 % (v/v) LCB-RM, respectively.

**Fig. S5: TNF-α activates inflammatory gene expression in differentiated adipocytes.** The cells were treated with the indicated concentration of TNF-α for 3 hours. **A** and **B** show the IL-6 and MCP-1/CCL2 gene expression levels, respectively.

**Original Western blot pictures**

**Except for Fig. 5B, Fig. 7A and Fig. 7F which have their own explanation:** These figures display the full original blots. The PVDF membrane was cut in two pieces so that the Western blots using anti-IκBα and anti-β-actin antibodies could be performed in parallel. The membrane was exposed to an X-ray film. The anti-IκBα and β-actin antibodies are very specific. Thus, the results are clear. Some of these figures have more than 1 exposures. However, the chosen results/areas for publication are boxed. Details for each lane are the same as described in its figure legend.


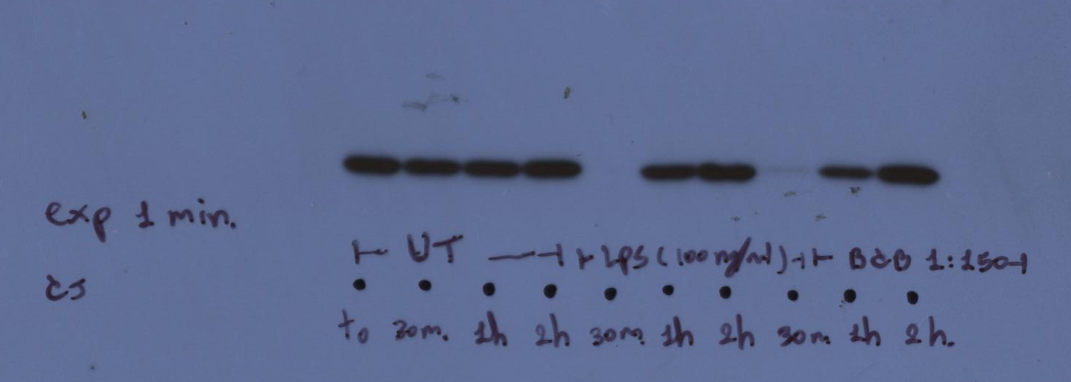
Fig. 5A, IκBα


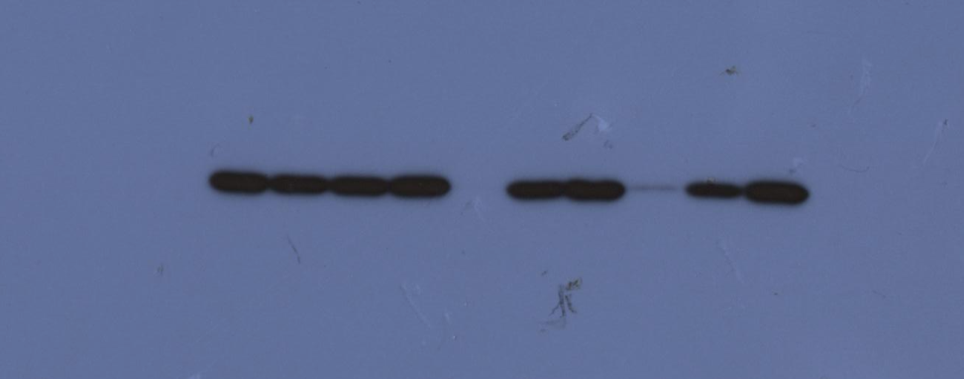


Fig. 5A, β-actin


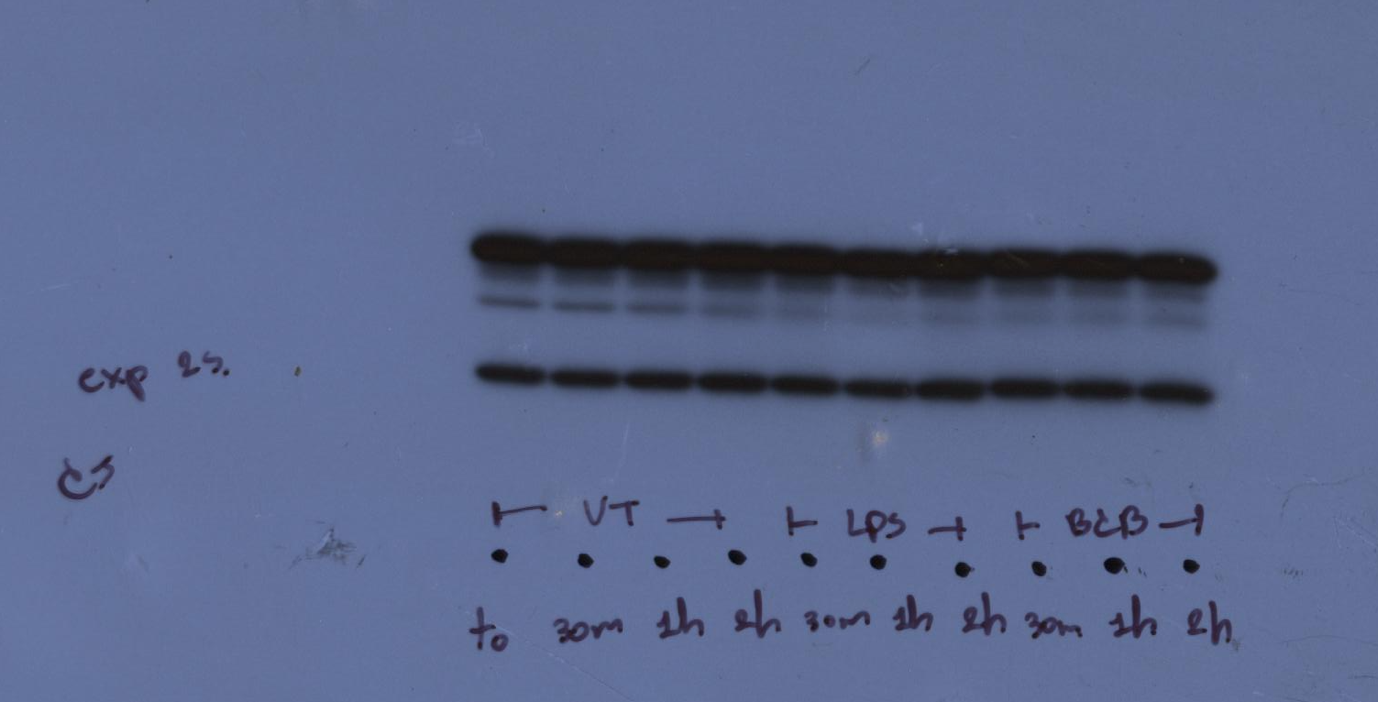


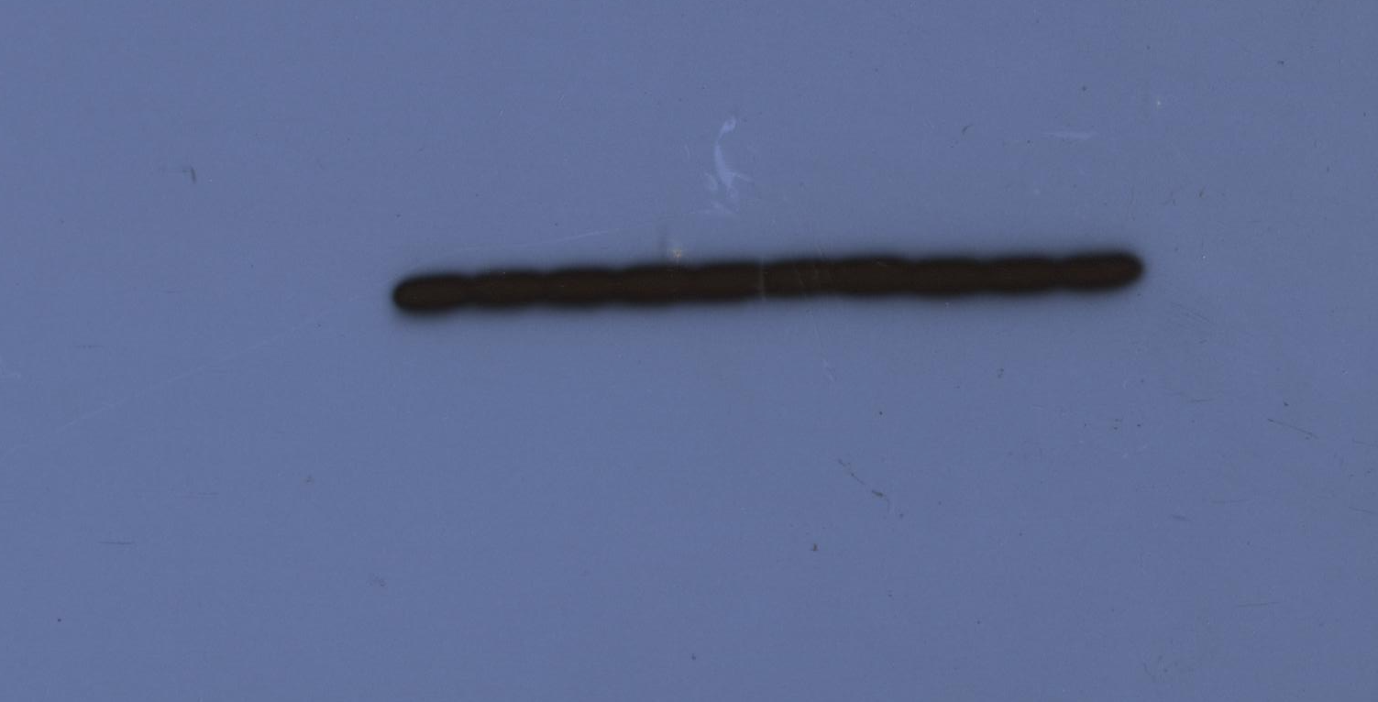


Fig. 5C, IκBα


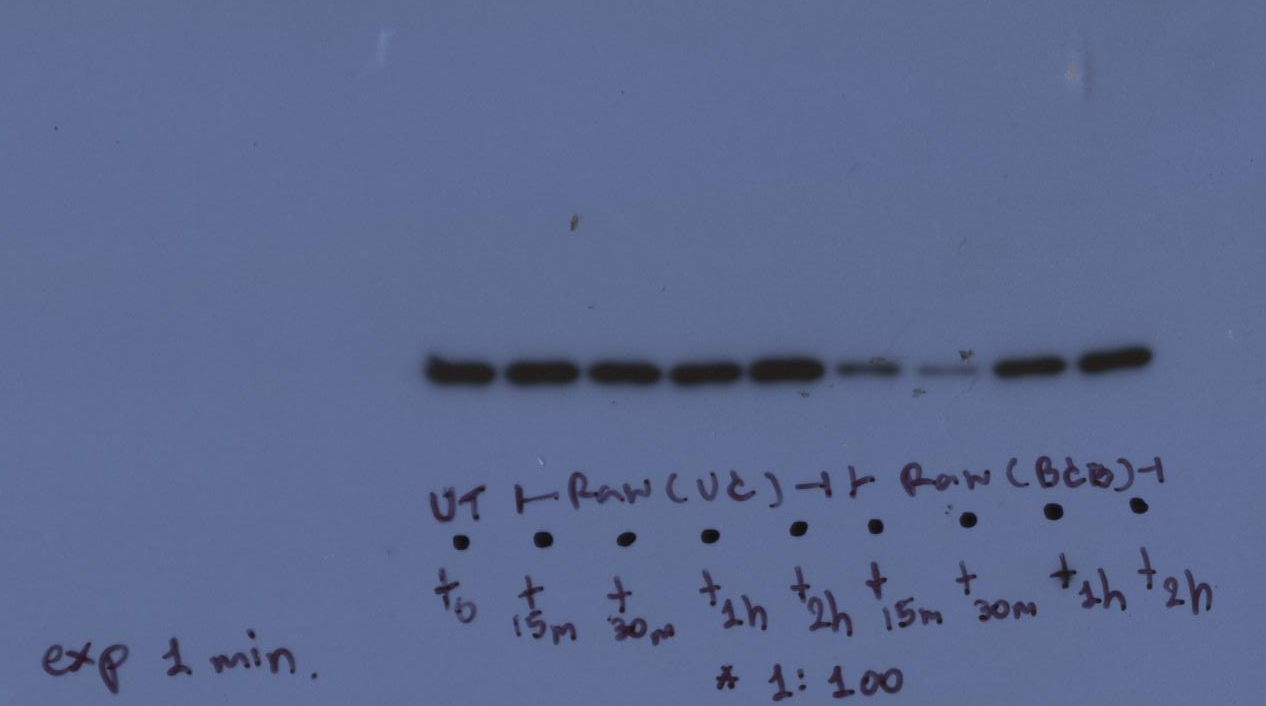


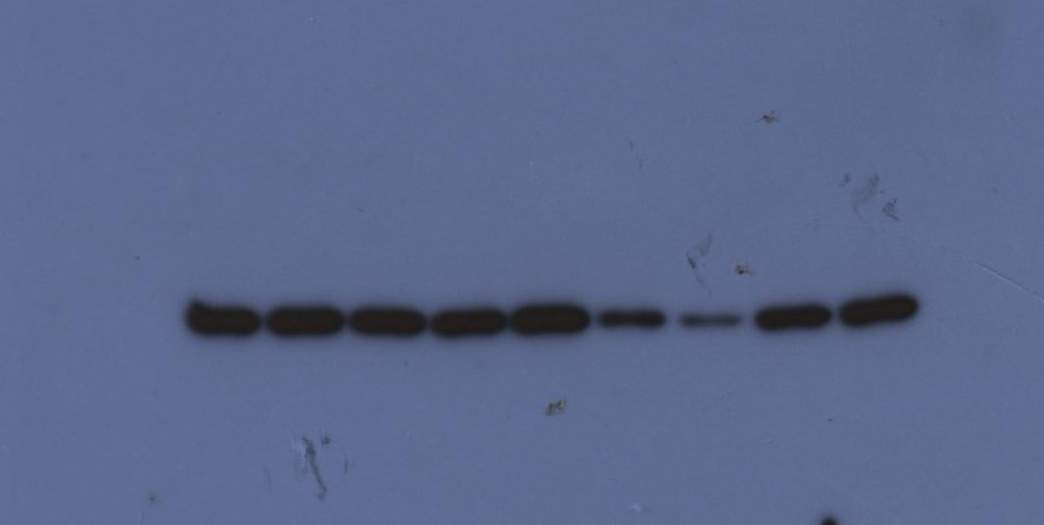


Fig. 5C, β-actin


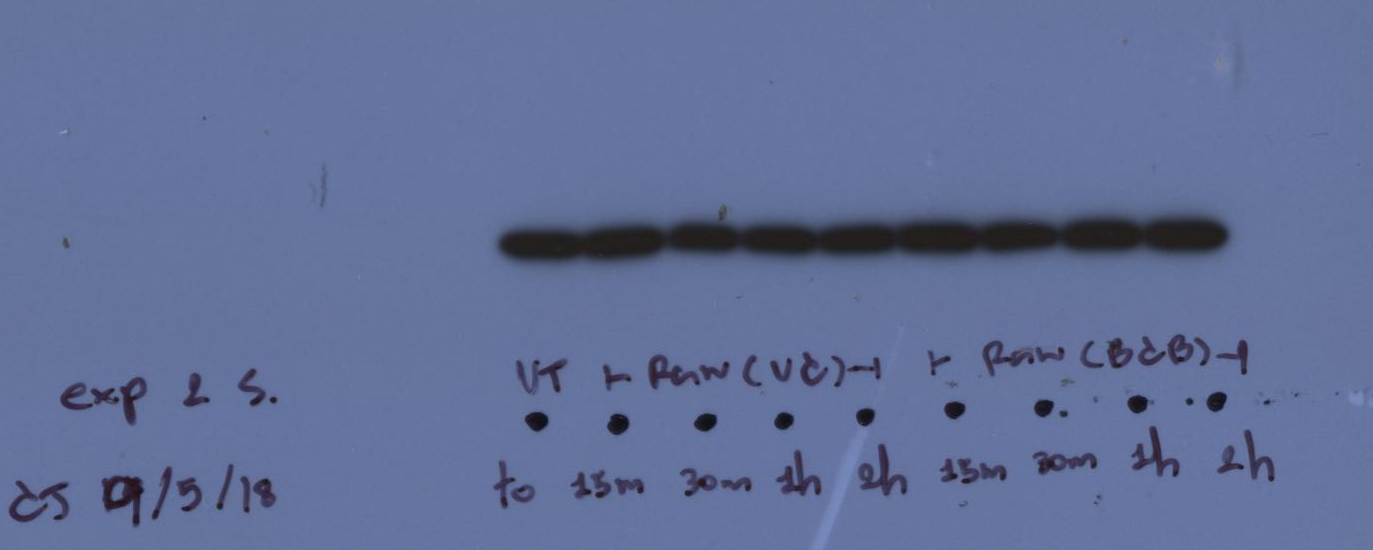


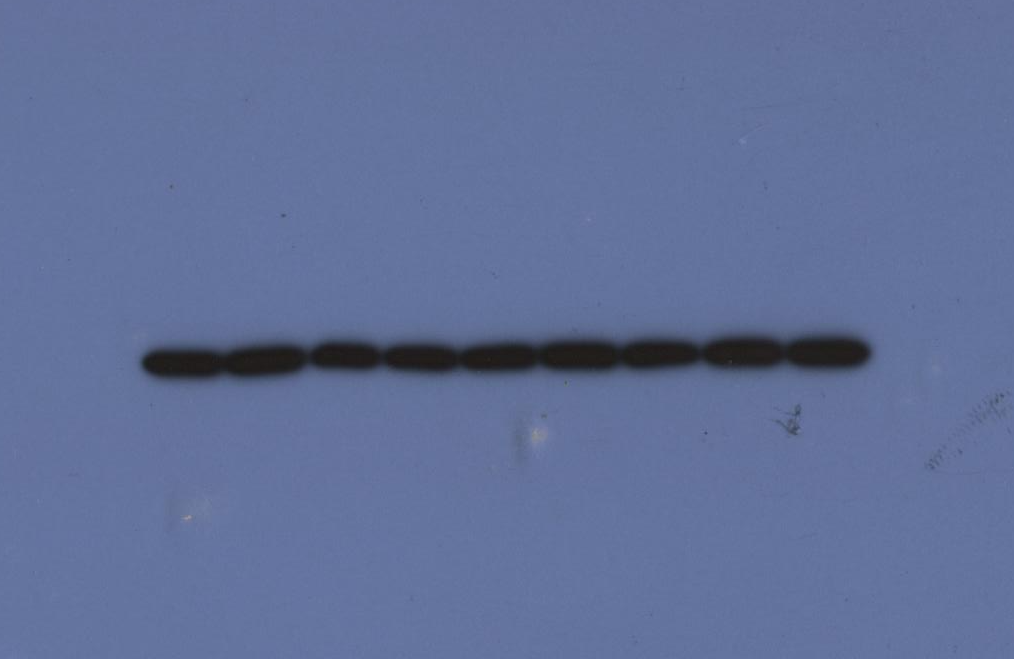


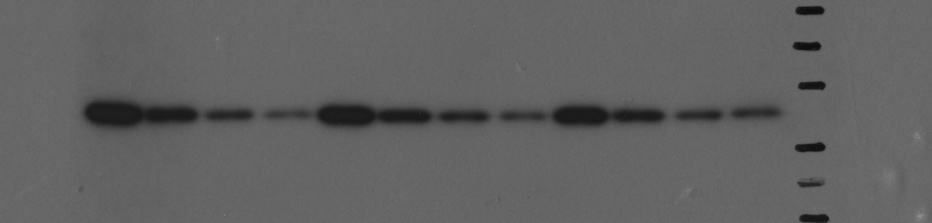


Fig. 5B, IκBα


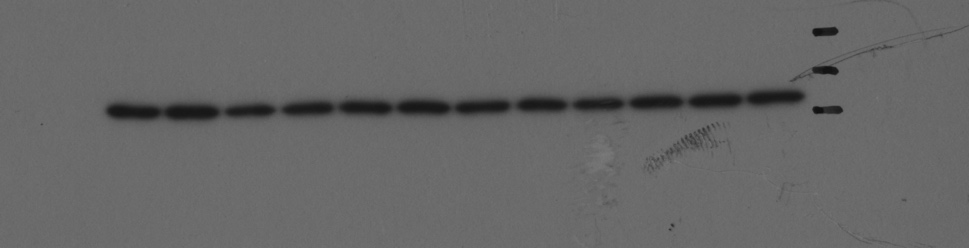
Fig. 5B, β-actin

**Explaination:** These figures display the full original blots for Fig. 5B. The PVDF membrane was cut in two pieces so that the Western blots using anti-IκBα and β-actin antibodies could be performed in parallel. In this gel, the triplicate of the experiments were loaded. Thus, the first 4 lanes from left to right are the first set, the next 4 lanes are the second set, and the last 4 lanes are the third set. Areas presented in the manuscript figures are boxed. The anti-IκBα and anti β-actin are very specific. Thus, they produce clear results. The details for each lane are the same as described for the Fig. 5B.


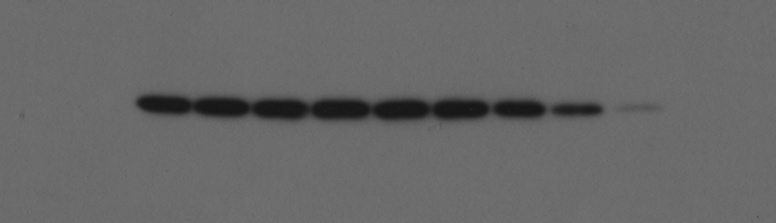
Fig. 5D, IκBα


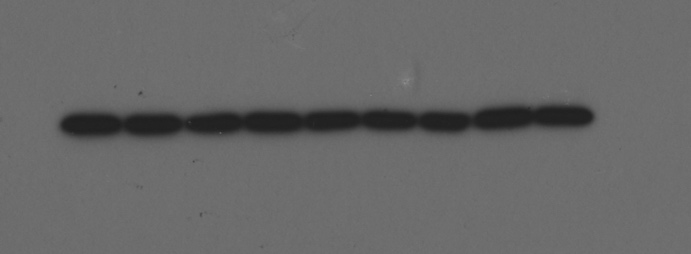


Fig. 5D, β-actin

Fig. 6A, IκBα


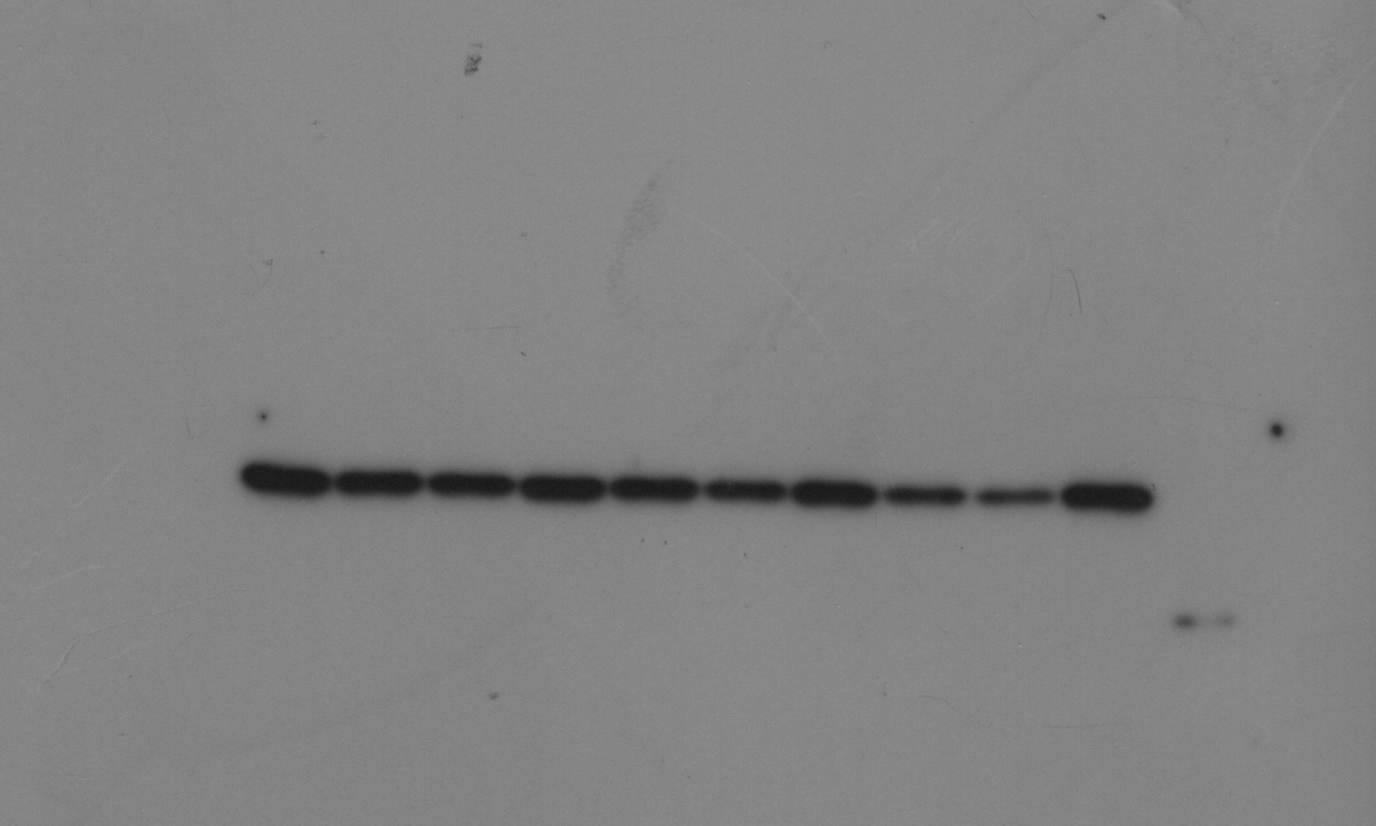


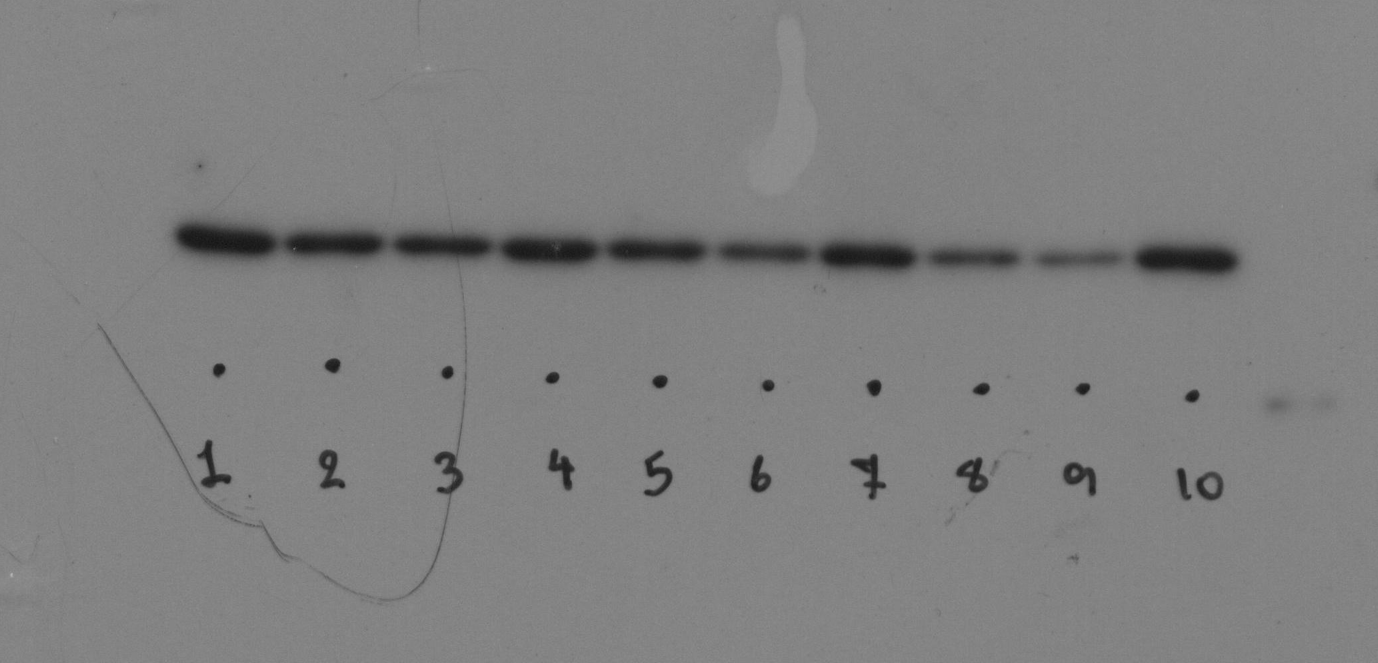


Fig. 6A, β-actin


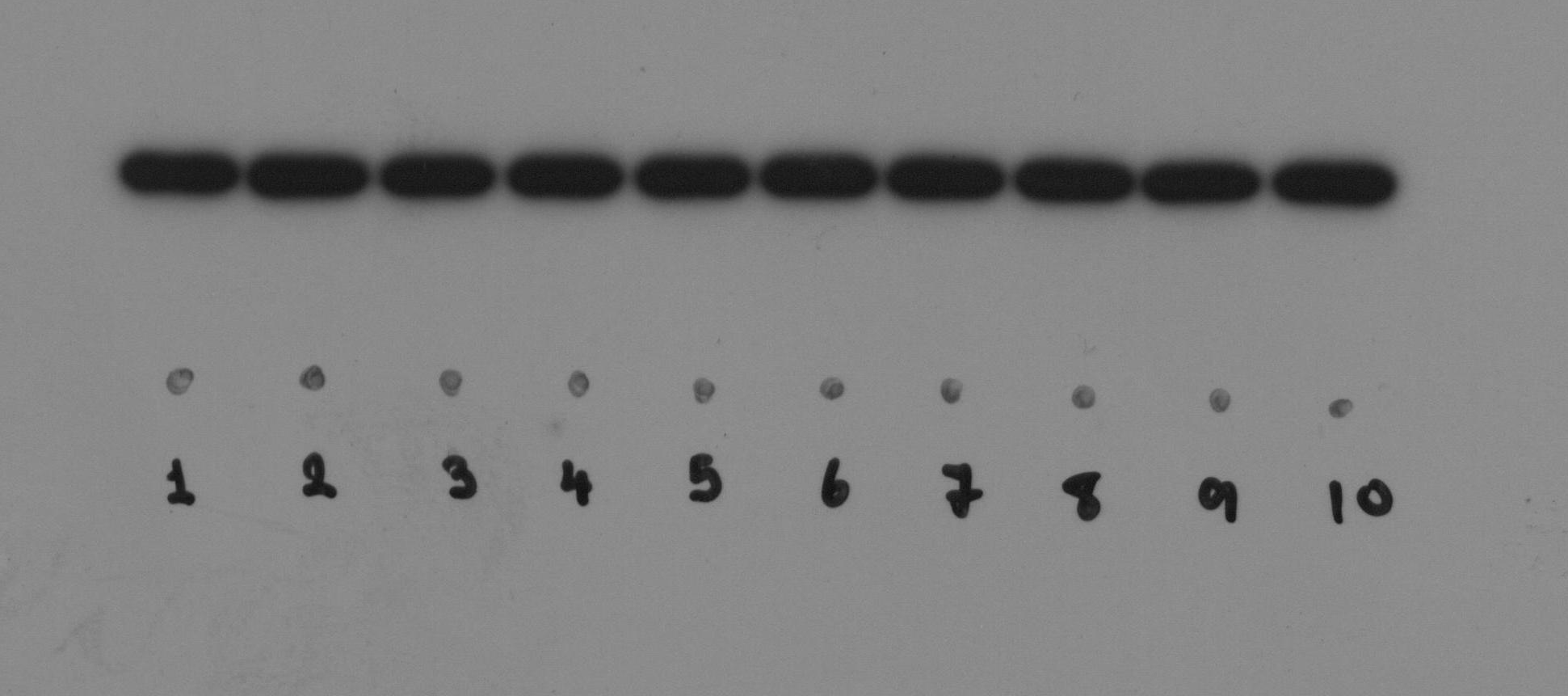


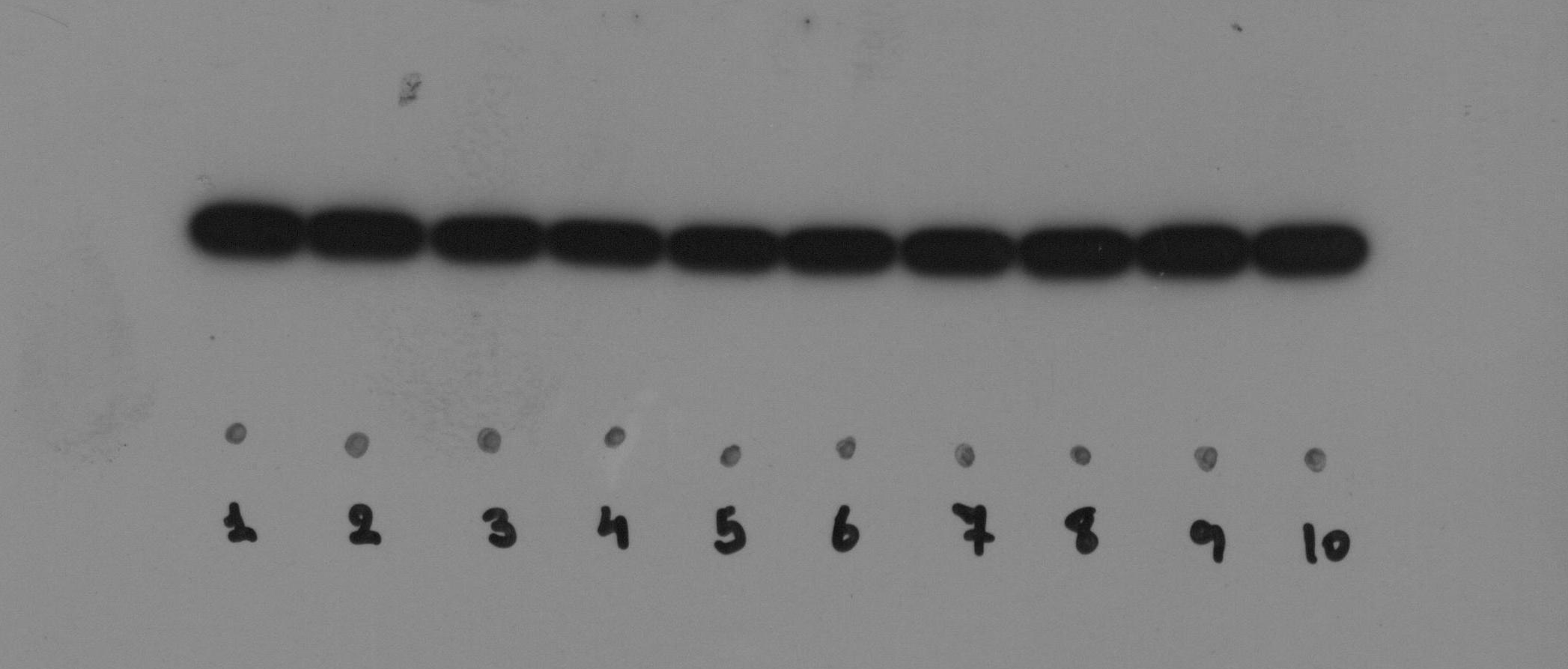


Fig. 6B, IκBα


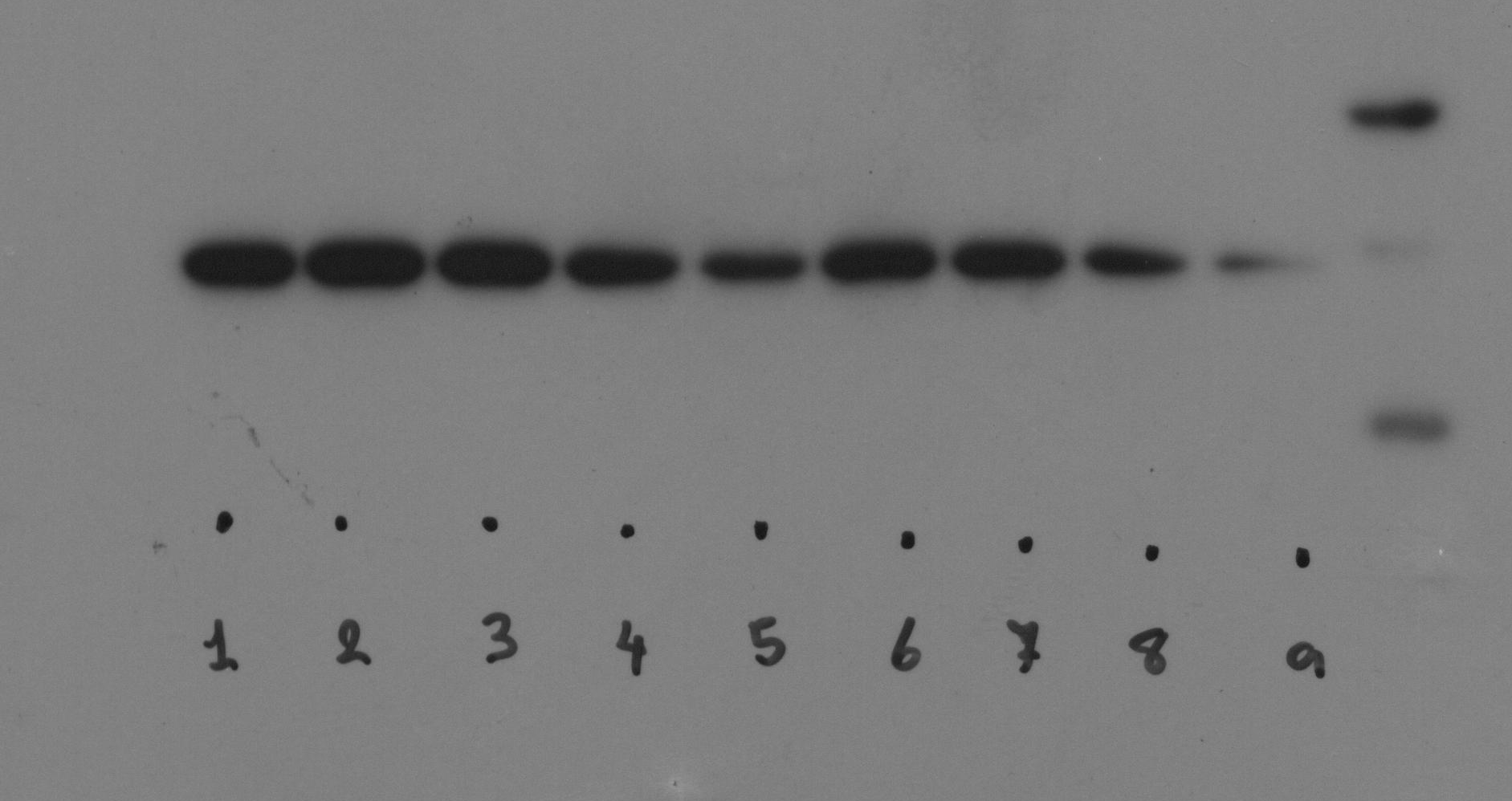


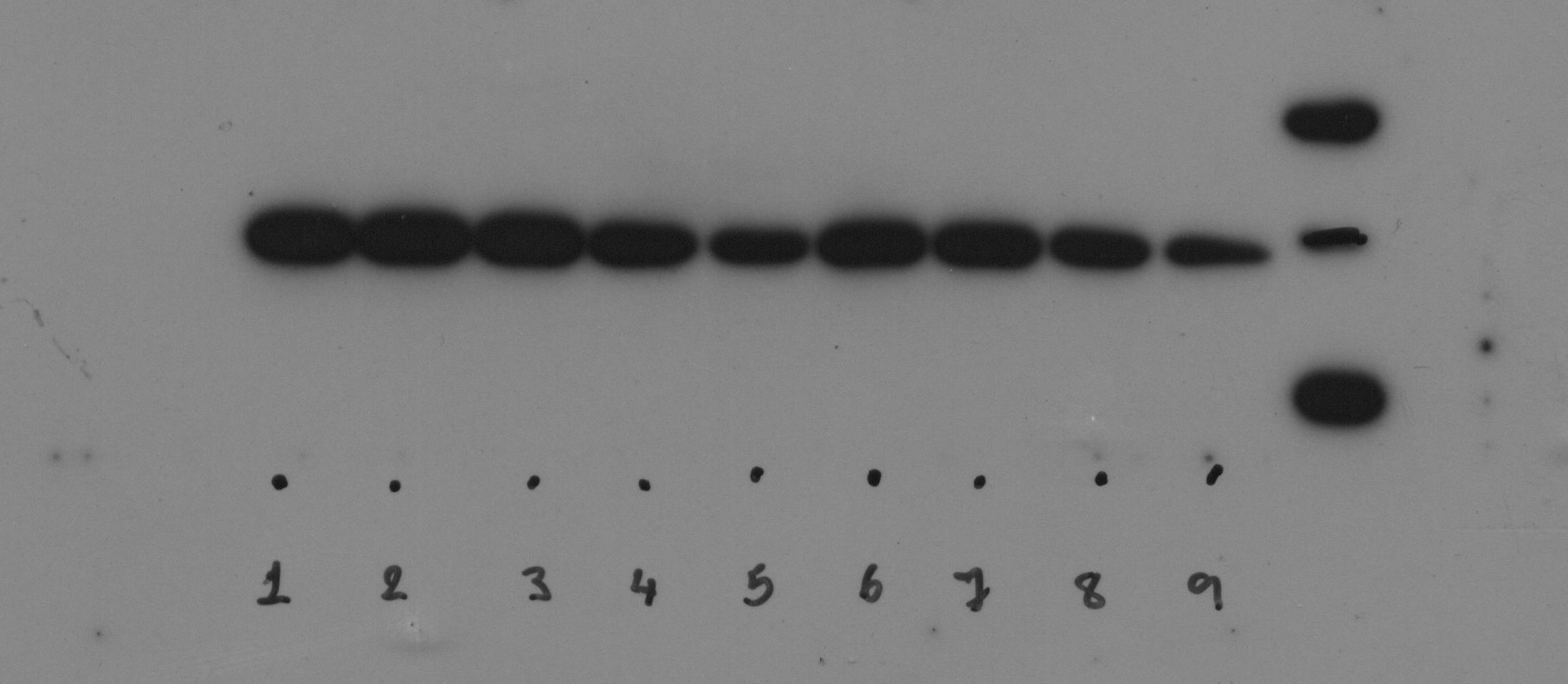


Fig. 6B β-actin


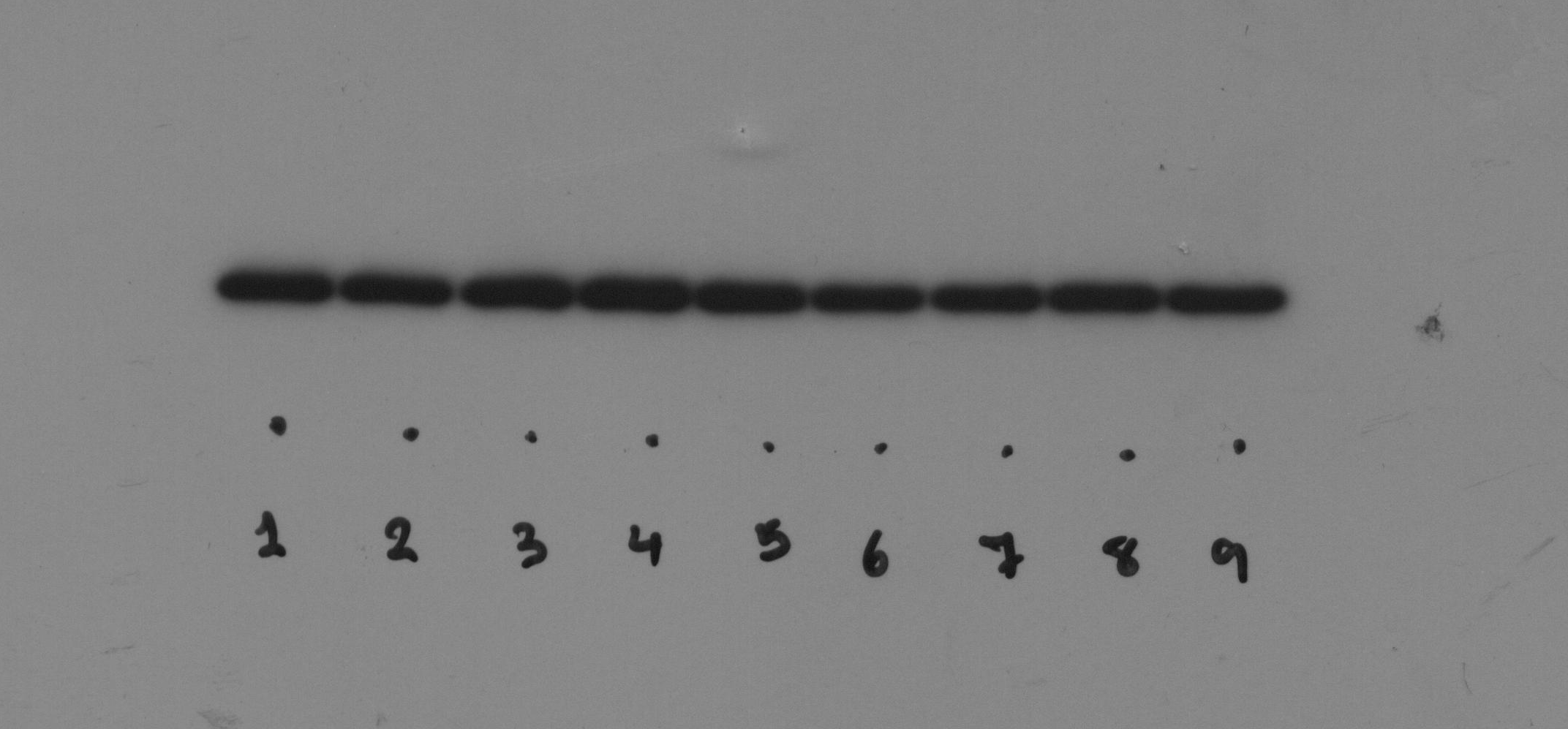


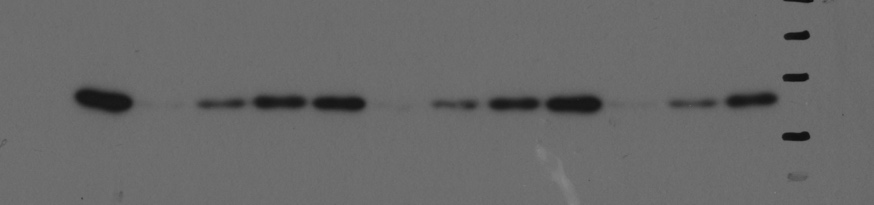
Fig. 7A, IκBα


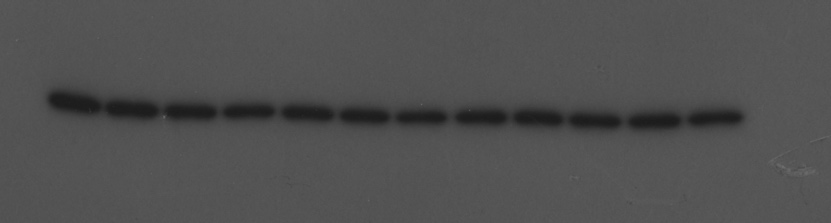


Fig.7A, β-actin

**Explaination:** These figures display the full original blots for Fig. 7A. The PVDF membrane was cut in two pieces so that the Western blots using anti-IκBα and β-actin antibodies could be performed in parallel. In this gel, the triplicate of the experiments were loaded. Thus, the first 4 lanes from left to right is the first set, the next 4 are the second set, and the last 4 are the third set. Areas presented in the manuscript figures are boxed. The antibodies for IκBα and β-actin are very specific. The details for each lane are the same as described for the Fig. 7A.


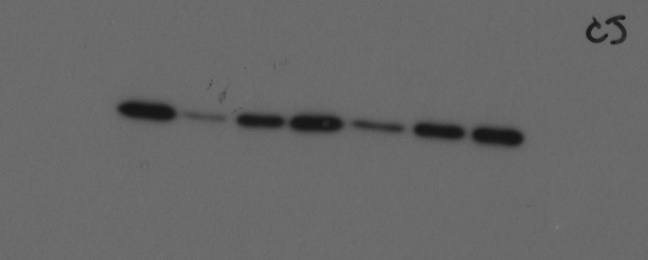


Fig. 7F, IκBα


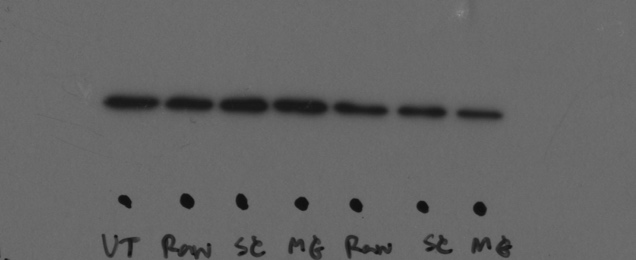
Fig. 7F, β-actin

**Explaination:** These figures display the full original blots for Fig. 7F. The PVDF membrane was cut in two pieces so that the Western blots using anti-IκBα and β-actin antibodies could be performed in parallel. Areas presented in the manuscript figures are boxed. The antibodies for IκBα and β-actin are very specific. The details for each lane are the same as described for the Fig. 7F.
